# Weighted Gene Co-Expression Network Analysis Combined with Machine Learning Validation to Identify Key Modules and Hub Genes Associated with SARS-CoV-2 Infection

**DOI:** 10.3390/jcm10163567

**Published:** 2021-08-13

**Authors:** Hassan Karami, Afshin Derakhshani, Mohammad Ghasemigol, Mohammad Fereidouni, Ebrahim Miri-Moghaddam, Behzad Baradaran, Neda Jalili Tabrizi, Souzan Najafi, Antonio Giovanni Solimando, Leigh M. Marsh, Nicola Silvestris, Simona De Summa, Angelo Virgilio Paradiso, Vito Racanelli, Hossein Safarpour

**Affiliations:** 1 Student Research Committee, Birjand University of Medical Sciences, Birjand 9717853577, Iran; hasan.karami.ms3@gmail.com; 2 Laboratory of Experimental Pharmacology, IRCCS-Istituto Tumori Giovanni Paolo II, 70124 Bari, Italy; a.derakhshani@oncologico.bari.it; 3 Immunology Research Center, Tabriz University of Medical Sciences, Tabriz 516615731, Iran; behzad_im@yahoo.com (B.B.); jalili.t.neda@gmail.com (N.J.T.); najafysoozan69@yahoo.com (S.N.); 4 Department of Computer Engineering, University of Birjand, Birjand 9717434765, Iran; ghasemigol@birjand.ac.ir; 5 Cellular and Molecular Research Center, Birjand University of Medical Sciences, Birjand 9717853577, Iran; dr.m.fereidouni@gmail.com; 6 Cardiovascular Diseases Research Center & Department of Molecular Medicine, School of Medicine, Birjand University of Medical Sciences, Birjand 9717853577, Iran; moghaddam4@yahoo.com; 7 Pharmaceutical Analysis Research Center, Tabriz University of Medical Sciences, Tabriz 516615731, Iran; 8 Department of Biomedical Sciences and Human Oncology, University of Bari “Aldo Moro”, 70121 Bari, Italy; antoniogiovannisolimando@gmail.com (A.G.S.); n.silvestris@oncologico.bari.it (N.S.); 9 Ludwig Boltzmann Institute for Lung Vascular Research, Neue Stiftingtalstraße 6/VI, 8010 Graz, Austria; leigh.marsh@lvr.lbg.ac.at; 10 Medical Oncology Unit, IRCCS-Istituto Tumori “Giovanni Paolo II” of Bari, 70124 Bari, Italy; 11 Molecular Diagnostics and Pharmacogenetics Unit, IRCCS-Istituto Tumori ‘Giovanni Paolo II’, 70124 Bari, Italy; desumma.simona@gmail.com; 12 National Cancer Research Centre, Istituto Tumori G Paolo II, 70124 Bari, Italy; a.paradiso@oncologico.bari.it

**Keywords:** COVID-19, SARS-CoV-2, transcriptional profiling, WGCNA, bronchial epithelium cells, treatment, explainable artificial intelligence

## Abstract

The coronavirus disease-2019 (COVID-19) pandemic has caused an enormous loss of lives. Various clinical trials of vaccines and drugs are being conducted worldwide; nevertheless, as of today, no effective drug exists for COVID-19. The identification of key genes and pathways in this disease may lead to finding potential drug targets and biomarkers. Here, we applied weighted gene co-expression network analysis and LIME as an explainable artificial intelligence algorithm to comprehensively characterize transcriptional changes in bronchial epithelium cells (primary human lung epithelium (NHBE) and transformed lung alveolar (A549) cells) during severe acute respiratory syndrome coronavirus 2 (SARS-CoV-2) infection. Our study detected a network that significantly correlated to the pathogenicity of COVID-19 infection based on identified hub genes in each cell line separately. The novel hub gene signature that was detected in our study, including *PGLYRP4* and *HEPHL1*, may shed light on the pathogenesis of COVID-19, holding promise for future prognostic and therapeutic approaches. The enrichment analysis of hub genes showed that the most relevant biological process and KEGG pathways were the type I interferon signaling pathway, *IL-17* signaling pathway, cytokine-mediated signaling pathway, and defense response to virus categories, all of which play significant roles in restricting viral infection. Moreover, according to the drug–target network, we identified 17 novel FDA-approved candidate drugs, which could potentially be used to treat COVID-19 patients through the regulation of four hub genes of the co-expression network. In conclusion, the aforementioned hub genes might play potential roles in translational medicine and might become promising therapeutic targets. Further in vitro and in vivo experimental studies are needed to evaluate the role of these hub genes in COVID-19.

## 1. Introduction

Since December 2019, millions of people have been directly or indirectly affected by the severe acute respiratory syndrome coronavirus 2 (SARS-CoV-2) global pandemic. Until 12 February 2021, more than 107 million people had already been reported to be infected, with 2,373,733 mortalities (https://coronavirus.jhu.edu/map.html, accessed on 12 February 2021). SARS-CoV-2 is a single-stranded RNA virus that belongs to the Coronaviridae family and can infect mammals and birds [1]. Clinical researchers have defined COVID-19 as an acute respiratory tract infection with diverse symptoms, including fever, dry cough, fatigue, myalgia, conjunctivitis, and pneumonia. However, some patients develop severe illnesses, including pneumonia, acute respiratory distress syndrome, pulmonary edema, acute kidney injury, or quick multiple organ failure [2,3,4,5]. Despite the high infection and mortality rates, the host’s immune response to infection with SARS-CoV-2 remains poorly understood [6,7]. In multiple cases, the involvement of the lungs suggests viral dissemination after the initial infection. Viral RNA has been identified in symptomatic patients in the upper airways, with greater viral loads in nasal swabs than those collected from the throat [8]. Comparable viral loads have been observed in an asymptomatic patient, suggesting that the nasal epithelium is a significant portal for initial infection and may act as the main reservoir for viral dissemination across the respiratory mucosa, an essential viral transmission mediating locus. To improve diagnostic and therapeutic approaches, it is critical to recognize not only the cells and mechanisms that host viruses and enable viral replication, but also what contributes to the inflammation and pathogenesis of the disease.

Dysregulated host immune response and inflammatory cytokine production are believed to correlate with the severity of the disease and poor prognosis in two other coronavirus-related infections, SARS-CoV and MERS-CoV [9,10]. Nevertheless, the underlying molecular mechanisms of the anomalous inflammatory responses under SARS-CoV-2 infection are still unclear.

Since the COVID-19 outbreak, numerous transcriptomic studies of various specimens of COVID-19 patients alongside experimental models have been attempted to further elucidate the dynamics of the host immune response along with gene regulatory networks. In this regard, Xiong et al. conducted a transcriptome sequencing of the RNAs isolated from bronchoalveolar lavage fluid as well as peripheral blood mononuclear cell (PBMC) samples of COVID-19 patients. Their results revealed host inflammatory cytokine profiles to SARS-CoV-2 infection in patients and highlighted the association between COVID-19 pathogenesis and excessive cytokine releases such as *CCL2*/*MCP-1*, *CXCL10*/*IP-10*, *CCL3*/*MIP-1A*, and *CCL4*/*MIP1B* [11]. In another study, Ren et al. used single-cell RNA sequencing (scRNA-seq) analysis to evaluate the expression and distribution of angiotensin-converting enzyme 2 (ACE2) and type II transmembrane serine protease (TMPRSS) genes in kidney cell components. They found that podocytes and proximal straight tubule cells were potential host cells targeted by SARS-CoV-2, resulting in acute kidney injury (AKI) caused by the SARS-CoV-2-induced cytopathic effect [12]. Given the critical role of chromatin factors such as topoisomerase I in regulating the transcriptional response to SARS-CoV-2 infection, Ho et al. evaluated transcriptional and epigenetic changes in the human alveolar basal epithelial carcinoma (A549) cell line expressing the human SARS-CoV-2 entry receptor Ace2 (A549-ACE2) infected with SARS-CoV-2 followed by the depletion of topoisomerase I and discovered promising effects of epigenetic therapy in modifying aberrant genome restructuring and selective suppression of inflammatory and anti-viral genes involved in severe COVID-19 [13]. In an effort to investigate the repertoire of viral epitopes promoting T cell-mediated immunity, Weingarten-Gabbay et al. uncovered the stable representation of SARS-CoV-2 HLA-I peptides over the time course of infection and the probable immune evasion mechanisms of SARS-CoV-2 by interfering with the IFN signaling and HLA class I-mediated antigen processing of highly expressed SARS proteins, as shown by dynamic transcriptional profiling of SARS-CoV-2-infected human lung A549 cells [14]. Interestingly, gene expression profiling of SARS-CoV-2-infected A549-ACE2 cells treated with potent anti-viral compounds allowed the exploration of the cholesterol biosynthesis pathway as the key mechanism underlying the blocking of viral replication, which holds the promise of providing indications for capable biomarkers and therapeutic targets [15]. More recently, RNA-seq analysis of SARS-CoV-2-infected A549-ACE2 cell line treated with calcium-channel blocker, amlodipine, identified that pre-treatment with amlodipine would significantly up-regulate the cholesterol biosynthesis-related gene pathway, which results in a marked decrease in SARS-CoV-2 viral load [16].

Due to the large-scale transcriptome data produced by high-throughput technologies such as RNA-seq and microarray, new appropriate approaches are required to efficiently extract meaningful associations from highly multivariate datasets. Several bioinformatics tools and machine learning algorithms, such as support vector machines, random forests, and extremely randomized trees classifiers, have been used to better understand complex essential gene interactions involved in disease development [2,17]. One large-scale genome-wide association approach named weighted correlation network analysis (WGCNA) is a tool for exploring hidden relationships between gene modules and disease traits [18]. WGCNA has been widely applied to construct a scale-free network alongside especially identifying functional modules of highly correlated genes to provide unbiased targets for genetic testing and potential therapies and illustrate the unraveling complexities of the disease by efficiently analyzing gene expression datasets [19,20,21,22,23].

In this study, we applied weighted gene co-expression network analysis (WGCNA) as well as LIME as an explainable artificial intelligence algorithm to investigate the transcriptional changes in primary human lung epithelium (NHBE) and transformed lung alveolar (A549) cell lines after SARS-CoV-2 infection in the hope of shedding light on the pathogenesis of this virus.

## 2. Materials and Methods

### 2.1. Gene Expression Dataset Acquisition and Preprocessing

**Figure 1:**
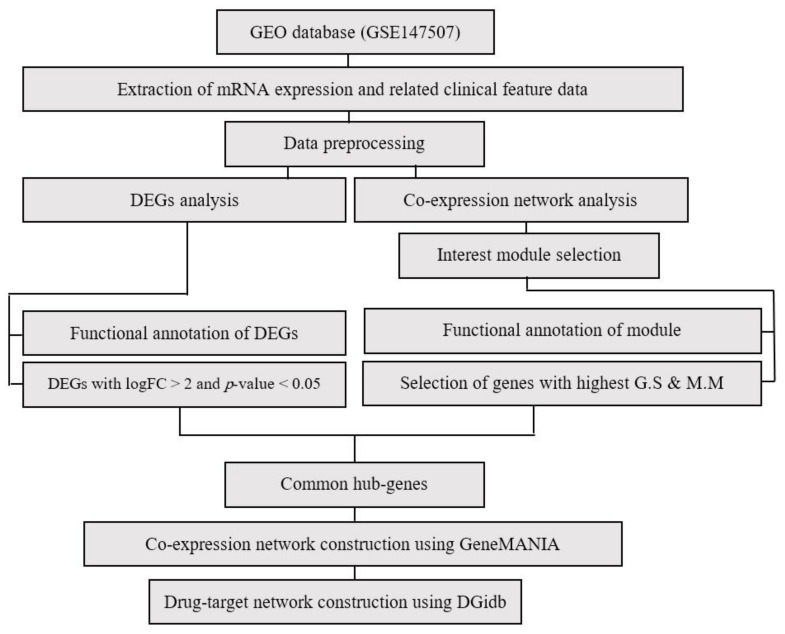
Flowchart of data preparation, processing, and analysis in this study.

Data analysis procedures are illustrated in Figure 1. The RNA-seq gene expression dataset GSE147507 (https://www.ncbi.nlm.nih.gov/geo/query/acc.cgi?acc=GSE147507, accessed on 8 March 2021) was obtained and downloaded from the Gene Expression Omnibus (GEO) database, which is based on Illumina NextSeq 500 platform. In total, the above dataset contains a raw read count of 20 samples of human lung epithelial cells, including independent biological triplicates of NHBE and A549 cells. The NHBE cells were mock-treated or infected with SARS-CoV-2 (USA-WA1/2020), and independent biological duplicates of A549 cells were mock-treated or infected with SARS-CoV-2, human respiratory syncytial virus (RSV), or seasonal influenza A virus (IAV) [24,25]. Following the mapping of the probe to gene IDs, the expression coefficient of variance was calculated to obtain a list with decreasing coefficient of variance for all processed expression data, and the first 4000 genes with a large coefficient of variance expression were selected.

### 2.2. Identification of Differentially Expressed Genes

Differentially expressed genes (DEGs) were considered by using the DESeq2 package v1.18.1. Genes were considered DEGs when they met the following criteria: FDR < 0.05, and |log_2_FC| ≥ 2.

### 2.3. WGCNA Network Construction and Identification of Significant Modules

Gene co-expression network of treated and control groups was reconstructed using WGCNA [26]. Briefly, the gene expression profile matrix was converted into the matrix of pairwise gene similarity according to the Pearson test, followed by conversion into the matrix of adjacency. According to the already represented scale-free gene co-expression topological algorithm, a minimum possible β value was considered so that the adjacency matrix could meet the scale-free topology criteria. For the next step, the topological overlap matrix (TOM) and dissimilarity TOM (dissTOM) were created using TOM similarity and dissimilarity modules. Finally, a module identification was performed by the dynamic tree cut, while the minimum module size was defined as 30. Modules with high similarity scores were merged with a threshold of 0.25. Moreover, the values of gene significance (G.S) were used to calculate the association of individual genes with COVID-19. In addition, the Module Membership (M.M) was defined as the ME correlation as well as the gene expression profile for each module. If the G.S and M.M are strongly associated, it can be stated that the most important (central) elements in the modules are also closely related to the trait. They can also be used to create a network and identify the hub genes.

### 2.4. Explain the Gene Importance by SP-LIME

Explainable AI (XAI) methods allow us to explain and interpret the result of machine learning models and reveal the importance of features in the model predictions [27,28]. LIME is an XAI algorithm that can explain the predictions of any classifier or regressor in a faithful way by approximating it locally with an interpretable model. Although an explanation of a single prediction provides some understanding of the reliability of the classifier to the user, it is not sufficient to evaluate and assess trust in the model as a whole. An extension of LIME is Submodular Pick LIME (SP-LIME), which selects a set of representative instances with explanations to denote the global importance of features in the explanation space via submodular optimization [29]. Algorithm 1 explains the SP-LIME procedures in more detail.
**Algorithm 1**: SP-LIME algorithm.Inputs:
S: The set of samples
F: The set of features
X: The set of instances for explanations
Run the explanation model on all instances $x_{i}\in X$ with the aid of the LIME algorithm.Construct the explanation matrix $W_{ij}$ that represents the local importance of the interpretable features for each instance.Compute the global importance of each feature $f_{j}\in F$ with $I_{j}=\overset{n}{\underset{i=1}{\sum }}\left|W_{ij}\right|$Maximize the coverage function by iteratively adding the instance with the highest maximum coverage gain.
Outputs:
Feature importance $I_{j}$
Instances that cover the important features V⊆S


### 2.5. Functional Enrichment of Significant Modules

The ClueGO (version 2.2.5) Plug-in tool on Cytoscape (version 3.6.0) was used to identify and visualize the enriched Gene Ontology (GO), KEGG pathway, and biological pathways in interesting gene modules (kappa score = 0.4).

### 2.6. Hub Gene Detection and Co-Expression Network Reconstruction

Differentially expressed genes with the highest G.S and M.M were chosen as hub genes. Venn diagram was generated using the freely available “Venny” v 2.1 software (http://bioinfogp.cnb.csic.es/tools/venny/, accessed on 7 March 2021). Functional networks were constructed by GeneMANIA (https://genemania.org, accessed on 7 March 2021) and visualized using Cytoscape v 3.0 software [30].

### 2.7. Evaluation of Selected Hub Genes’ Behavior in Other Virus-Based Infections

To evaluate the expression behavior of selected hub genes in other virus-based infections, including SARS-CoV-2, RSV, IAV, and IAV-ΔNS1, we performed DEG analysis on related datasets from GSE147507.

### 2.8. Evaluation of Selected Hub Genes’ Behavior in Lung Tissues and Secretions Isolated from COVID-19 Patients

To further characterize selected gene signatures’ expression level changes in respiratory specimens extracted from COVID-19 patients, we performed DEG analysis on related datasets including GSE147507, GSE163426, GSE150316, GSE163151, and GSE154770.

### 2.9. Evaluation of Selected Hub Genes’ Behavior in COVID-19 Patients with Severe Pneumonia Varied by Viral Load

In order to illustrate the hub gene expression differences between lung samples by viral load strata, we used GSE150316, which included postmortem lung sections of individuals who succumbed to severe SARS-CoV-2 infection with definitive viral load labels. Additionally, transcriptomic changes in selected hub genes during disease resolution and viral clearance were investigated using DEG analysis on GSE154770.

### 2.10. Identification of Candidate Regulatory Drugs

The firmly established Drug–Gene Interaction Database (DGIDB) (http://www.dgidb.org, accessed on 15 March 2021) was used to predict functional and drug-able hub genes with the list of commercially available or clinical trial drugs [31].

## 3. Results

### 3.1. Preprocessing and DEG Analysis

**Figure 2:**
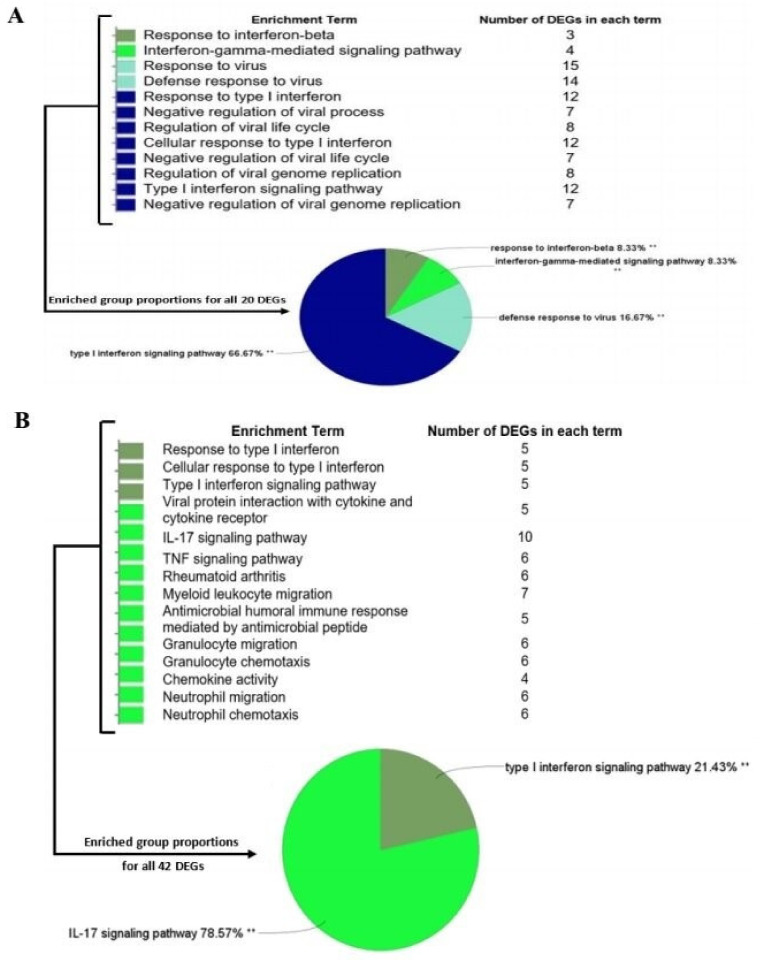
DEG characterization. Biological process identification and KEGG analysis of A549 and NHBE DEGs (A549 (**A**) and NHBE (**B**) cell lines). ** *p*-value < 0.01.

We performed quantile normalization to reduce the effects of technical noises. No outliers were observed in dataset samples by sample clustering. Thus, all samples were included in the analysis (Figure S1). Based on DEG analysis between SARS-CoV-2-treated and mock-treated cell lines, there were a total of 20 genes in A549 cells (all up-regulated) and 42 genes in NHBE cells (34 up-regulated, eight down-regulated) (Figure S2). The DEGs were then selected for subsequent analysis. The GO and KEGG pathway analysis of DEGs is represented in Figure 2.

### 3.2. Identification of WGCNA Modules

Based on the variance of expression values, a total of 4000 genes were included in WGCNA. Afterward, the β value equal to 12 and 24 was considered for A549 and NHBE cells, respectively, and a weighted gene co-expression network of treated and mocked samples was reconstructed (Figure S3). The result of the dynamic tree cut gave a total of 20 co-expressed modules recognized by a hierarchical clustering dendrogram with a range size of 30 (mediumpurple2) to 1313 (brown4) in the A549 dataset and 24 co-expressed modules with a range size of 33 (yellow4) to 841 (darkolivegreen) in the NHBE dataset (Figure S4).

### 3.3. Module–Trait Association Analysis and Functional Annotation Analysis of Interesting Modules

The evaluation of the relationship between each module eigengene and the trait and module–module association recognized the Turquoise module (r = 1, *p*-value = 1.00 × 10^−5^) in the A549 cell type (Table S1 and Figures S5 and S6).

**Figure 3:**
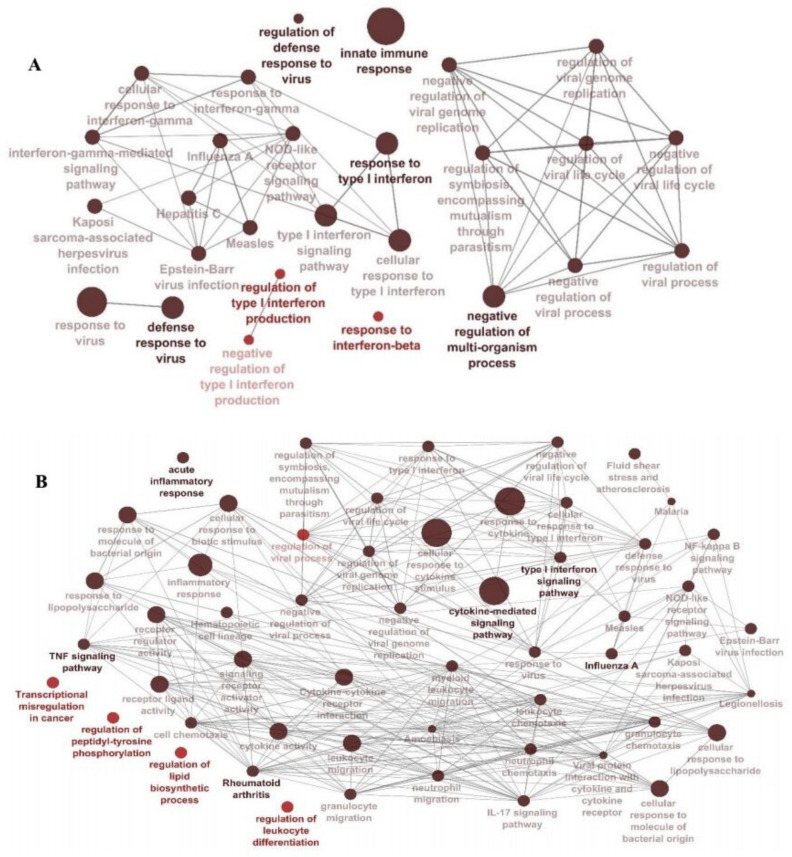
Biological processes and pathways detected within the (**A**) Turquoise module from the A549 dataset and (**B**) Yellow-green module from the NHBE dataset. Gene Ontology and pathway analysis was performed using significant genes across all datasets. Node size represents the counts (number of genes that are part of each pathway), and node color corresponds to the statistical significance. The darker the pathway node, the more statistically significant it is, with a gradient from red (*p*-value 0.05–0.005) to black (*p*-value < 0.0005).

Gene Ontology (GO) and functional and KEGG pathway enrichment analysis were conducted to explore the meaningful biological relevance of the interesting modules using ClueGO (Figure 3). As shown in Figure 3, functional annotations of the Turquoise (A549) and Yellow-green (best module of NHBE ((Table S2), Figure S6)) showed mainly similar enrichments and focused on the immune responses against viral infection. Common pathways were: regulation of viral life cycle, negative regulation of viral genome replication, type 1 interferon signaling pathway, NOD-like receptor signaling pathway, defense response to viruses such as influenza A, measles, Herpes simplex 1 virus, and Epstein–Barr virus.

### 3.4. Hub Gene Identification and Network Analysis of Interesting Modules

**Figure 4:**
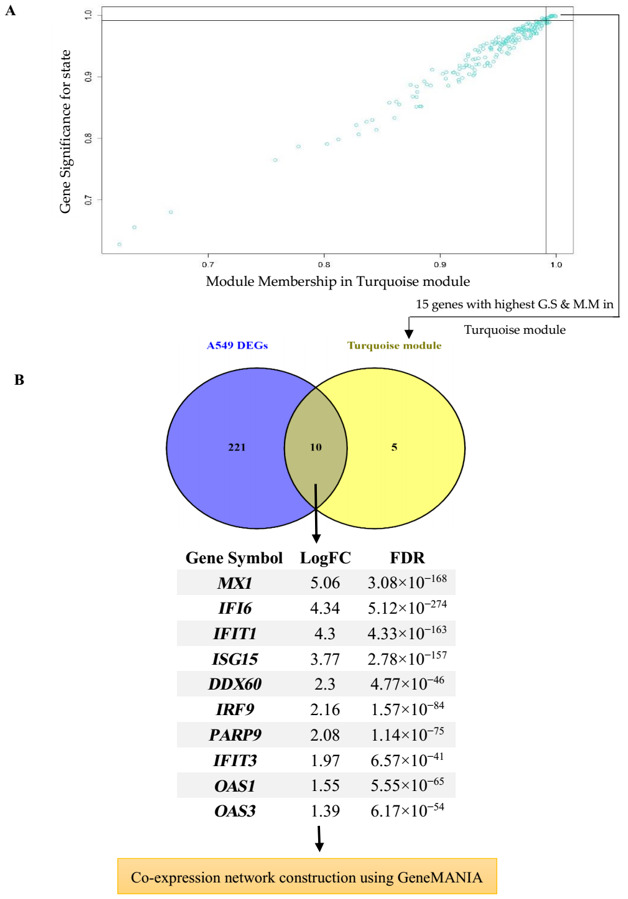
Hub gene detection for the A549 dataset. (**A**) Turquoise module features of G.S and M.M significantly correlated with the SARS-CoV-2 trait (mock-infected and SARS-CoV-2-infected). Each point represents an individual gene within each module, which is plotted by G.S on the y-axis and M.M on the x-axis; (**B**) Evaluation of similarities between DEG and hub gene lists using a Venn diagram. Ten genes that were similar in both lists were then imported to GeneMANIA to construct a co-expression network.

**Figure 5:**
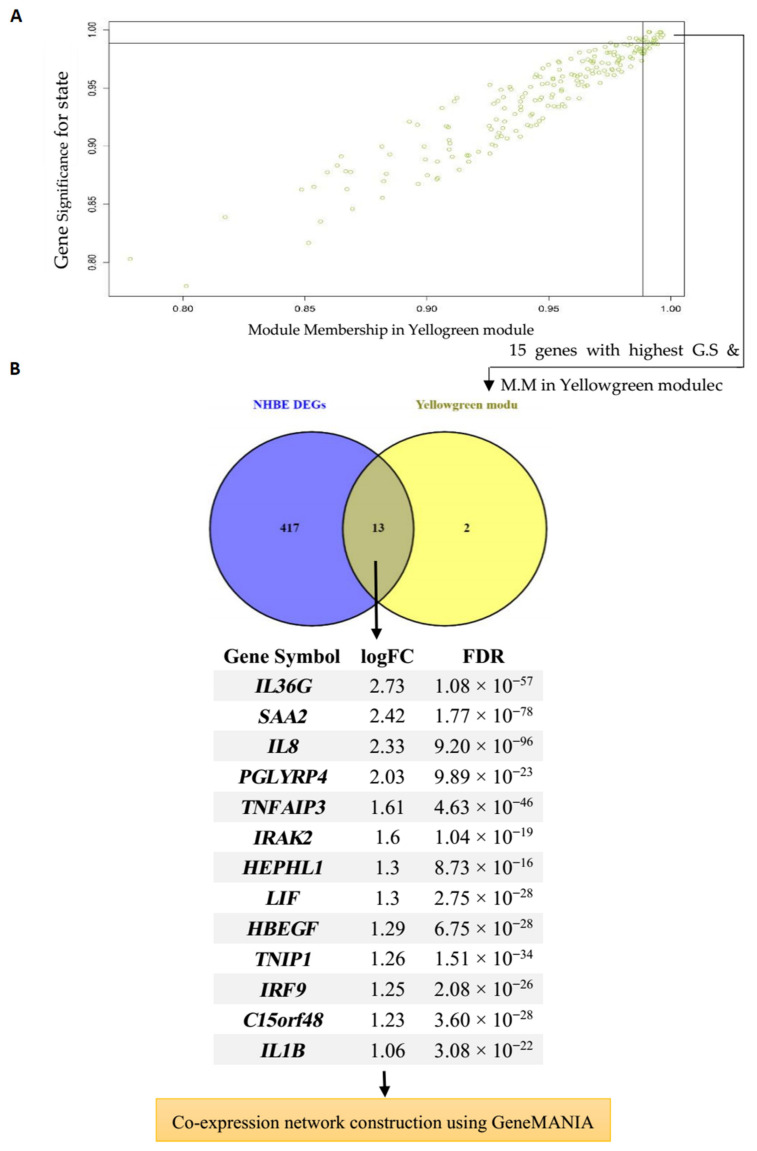
Hub gene detection for NHBE dataset. (**A**) Yellow-green module features of G.S and M.M significantly correlated with the SARS-CoV-2 trait (mock-infected and SARS-CoV-2-infected). Each point represents an individual gene within each module, which is plotted by G.S on the y-axis and M.M on the x-axis; (**B**) Evaluation of similarities between DEG and hub gene lists using a Venn diagram. Thirteen genes that were similar in both lists were then imported to GeneMANIA to construct a co-expression network.

The correlation between the features (M.M and G.S) of the selected modules led to the detection of interesting hub genes that were highly associated with the pathogenesis of the disease (Figure 4A and Figure 5A). The 15 genes with maximum M.M and G.S scores in each module were then compared to identify feature genes by DEG analysis, and overlapping genes were considered as final hub genes (Figure 4B and Figure 5B). For the Turquoise module, these genes included *OAS1* (2′-5′-Oligoadenylate Synthetase 1), *OAS3* (2′-5′-Oligoadenylate Synthetase 3), *MX1* (Myxovirus (Influenza) Resistance 1), *IFIT1* (Interferon Induced Protein with Tetratricopeptide Repeats 1), *IFIT3* (Interferon-Induced Protein With Tetratricopeptide Repeats 3), *ISG15* (ISG15 Ubiquitin Like Modifier), *IFI6* (Interferon alpha-inducible protein 6), *DDX60* (DExD/H-Box Helicase 60), *IRF9* (Interferon Regulatory Factor 9), and *PARP9* (Poly(ADP-Ribose) Polymerase Family Member 9), and for the Yellow-green module *IRF9*, *PGLYRP4* (Peptidoglycan Recognition Protein 4), *IL36G* (Interleukin 36 Gamma), *SAA2* (Serum Amyloid A2), *C15orf48* (Chromosome 15 Open Reading Frame 48), *TNFAIP3* (TNF Alpha Induced Protein 3), *TNIP1* (*TNFAIP3* Interacting Protein 1), *HEPHL1* (Hephaestin-Like Protein 1), *IRAK2* (Interleukin-1 Receptor-Associated Kinase-Like 2), *HBEGF* (Heparin Binding EGF Like Growth Factor), *LIF* (LIF Interleukin 6 Family Cytokine), *IL1B* (Interleukin 1 Beta), and *IL-8* (Interleukin 8) were the hub genes that had a high significance for immune-related COVID-19 response. The critical role of these genes in the host response to various other viruses has been described by many studies [32,33,34,35,36,37,38,39,40,41,42].

### 3.5. SP-LIME as an Explainable AI Method for Identifying Important Genes

**Figure 6:**
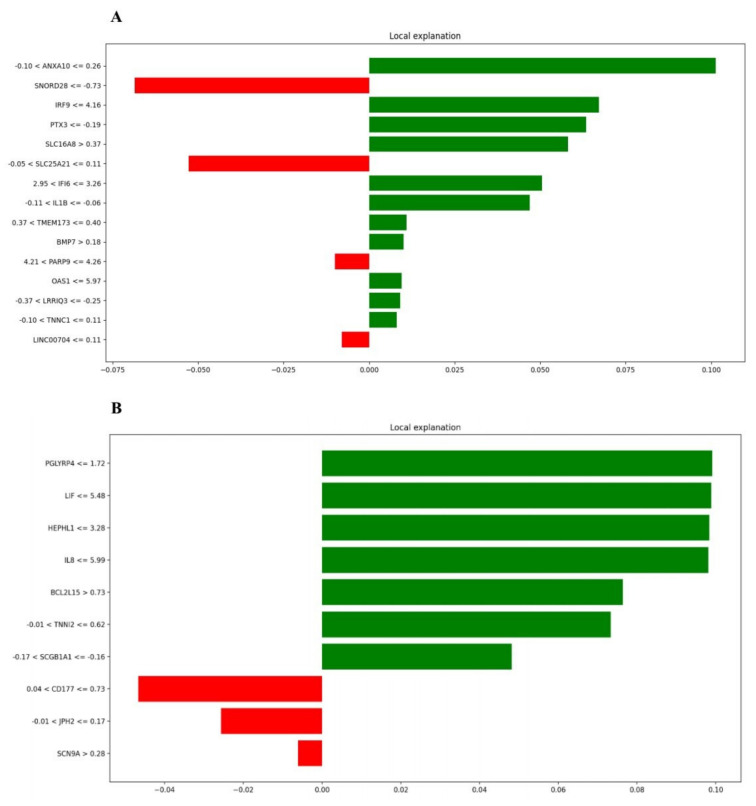
Top genes and their contributions in the model predictions by the SP-LIME algorithm. (**A**) Turquoise module A549 cell line; (**B**) Yellow-green module from NHBE cell line.

In this paper, we applied the SP-LIME algorithm to detect the importance of genes in the model predictions. We applied multiple classifiers to the collected dataset and ran the SP-LIME algorithm to explain their results. Figure 6 shows the experiments of the SP-LIME algorithm with the random forest classifier, presenting the genes with the highest impact on model prediction. For example, *PGLYRP4* and *ANXA10* are the most critical genes in the classification process. Interestingly, some feature genes were screened out based on the machine learning algorithm, including *IRF9*, *IFI6*, *OAS1*, *PARP9*, *PGLYRP4*, *LIF*, *HEPHL1*, and *IL8*, which were overlapped with identified hub gene candidates, highlighting the potential involvement of these hub genes during COVID-19 pathogenesis. The green and red colors shows the contribution of each gene to have a positive/negative impact for classifying samples towards the COVID-19 state.

### 3.6. Evaluation of Selected Hub Genes’ Behavior in Other Virus-Based Infections

In order to compare differences between SARS-CoV-2, RSV, IAV, and IAV-ΔNS1 molecular pathogenicity, we performed DEG analysis on 22 well-characterized hub genes (10 for A549 cells and 12 for NHBE cells).

As indicated in Table 1 there are no reliable data regarding IAV and IAV-ΔNS1 in the A549 dataset, but expression values in RSV and SARS-CoV-2 showed some critical information. In this regard, eight of ten hub genes in the A549 dataset, including *MX1*, *IFIT1*, *IFIT3*, *OAS1*, *OAS3*, *ISG15*, *DDX60*, and *PARP9*, have higher expression values in RSV compared to SARS-CoV-2. On the other hand, two out of ten hub genes, including *IFI6* and *IRF9*, showed higher expression values in SARS-CoV-2 infection.

Interestingly, the expression pattern between two lists of SARS-CoV-2 and RSV in A549 cells revealed a diminished anti-viral response to SARS-CoV-2, discriminating this response from common robust interferon induction of anti-viral genes, which had been reported in other studies after viral infections [43]. However, considering obvious suppressed expression levels of *IFN-1* and *IFN-3* by means of SARS-CoV-2, the underlying mechanism of this unexpected anti-viral response is still to be ascertained. In line with this, *IRF9* and *IFI6*, amongst these hub genes, are very attractive due to their unabated transcriptional response to SARS-CoV-2 when compared to RSV. Considering several recent studies that demonstrated the pivotal role of *IRF9* and *IFI6* in COVID-19 pathogenesis, these results support a model in which the unique unmuted expression of *IRF9* in the response of SARS-CoV-2 infection would be responsible for the observed significant up-regulation of at least a subset of interferon-stimulated genes (ISGs) to escape from robust control over IFN-1 and IFN-3 expression and strengthen the idea that *IFI6* up-regulation could be an effective anti-viral strategy to protect healthy respiratory epithelial cells from early apoptosis elicited by surrounding pro-apoptotic cytokines leading to enhanced IFN production [43,44].

The NHBE cell line showed informative data from SARS-CoV-2, IAV, and IAV-ΔNS1 with appropriate *p*-values. However, no evidence could help to compare the gene expression profile of SARS-CoV-2 and RSV in NHBE cells (Table 1).

In this study, the results showed that the fold change expression of *IFI6* in A549 cells was equal to 19.5, with a significant *p*-value of 5.12 × 10^−274^. Interestingly, the *IFI6* expression value was much lower in cells that were infected with RSV (7.5-fold with a *p*-value of 5.97 × 10^−43^) (Table 1).

### 3.7. Evaluation of Selected Hub Genes’ Behavior in Respiratory Specimens of COVID-19 Patients

**Figure 7:**
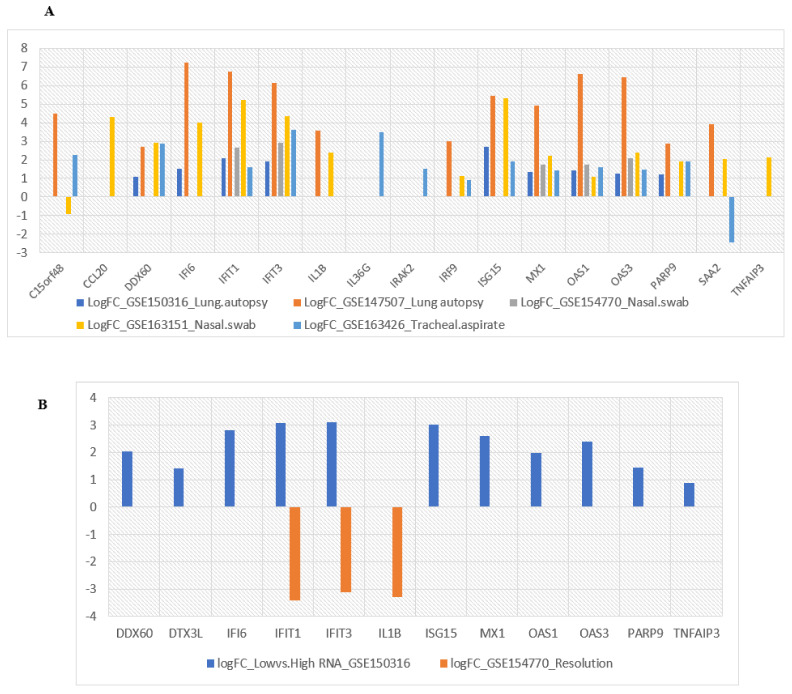
(**A**) Comparison of the expression level of selected hub genes in respiratory specimens of COVID-19 patients; (**B**) Evaluation of transcriptional changes in identified hub genes in samples with high versus low SARS-CoV-2 viral load alongside their differential expression over the course of SARS-CoV-2 infection.

The expression patterns of all 22 selected hub genes were investigated in five datasets related to respiratory specimens of COVID-19 patients to understand whether identified transcriptional signatures could be adequately validated by in vitro studies (Figure 7A). Consistent with our in vitro results, most of the selected hub genes displayed significant overexpression in lung and epithelial tissues. Notably, the expression levels of *SAA2* and *C15orf48* were significantly down-regulated in SARS-CoV-2-infected tracheal aspirate and nasal swab specimens, respectively, which might be candidates for further evaluations. In this regard, recent studies reported an impaired interferon signaling response in tracheal aspirates of patients with severe COVID-19, which may serve as a mechanism underlying reduced SAA2 stimulation in the lower respiratory tract of COVID-19 patients with ARDS [45,46].

### 3.8. Evaluation of Selected Hub Genes’ Behavior in High and Low Viral Load of Samples from SARS-CoV-2-Infected Patients

As indicated in Figure 7B, these findings further suggest a model of disease evolution in critically ill COVID-19 patients with pneumonia characterized by an early phase of robust IFN host response to the high replication of the virus followed by a later phase of viral clearance with the suppression of IFN signaling, as supported by previous studies [47]. In addition to IFN pathway genes, the higher anti-viral activity of hub genes related to TNF signaling and NAD metabolisms such as *TNFAIP3*, *PARP9*, and *DTX3L* would be able to characterize patients with a high viral load, indicating a clear association of their expression to the presence of the virus and the phase of the disease. Considering the predominant expression of *TNFAIP3* in the acute stage as compared to the convalescent stage of SARS-CoV-2 infection, promising repurposable drugs such as methotrexate, ustekinumab, and TNF-α inhibitors targeting *TNFAIP3* would likely be more potent in controlling COVID-19 hyper inflammation if administered in the early phase of the disease [48,49,50].

### 3.9. Drug–Target Network Construction

**Figure 8:**
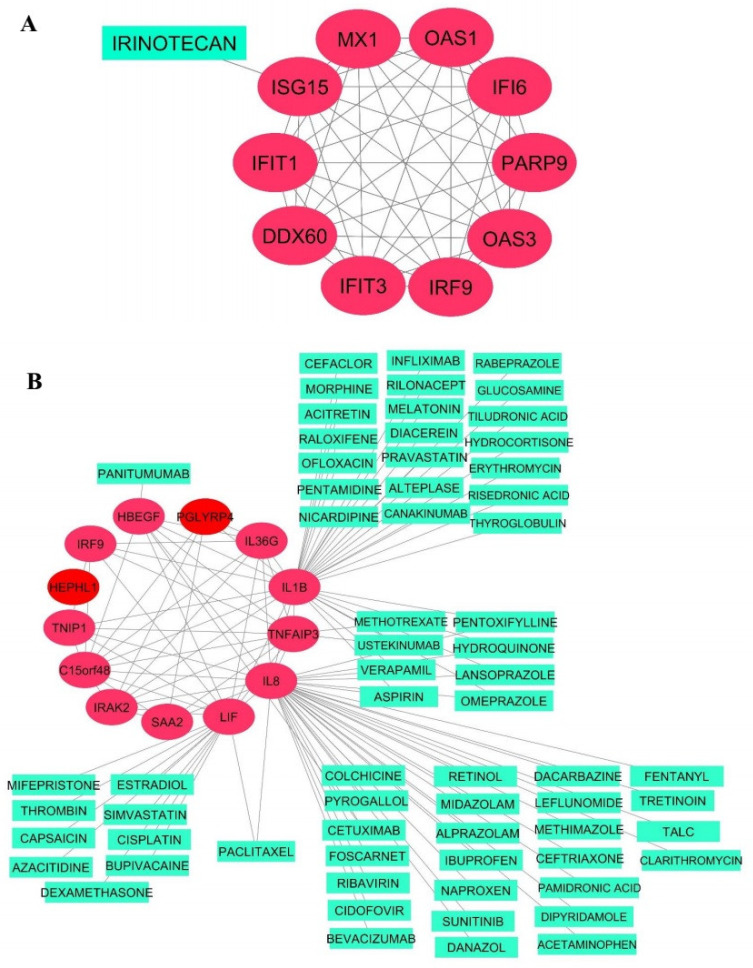
Co-expression network and related drugs of Turquoise (**A**) and Yellow-green (**B**) modules.

The two hub gene sets that met the above criteria were then separately imported to the GeneMANIA database to visualize interactions between the genes in each co-expression module. The resulting co-expression networks are visualized as red circles in Figure 8.

A drug repositioning analysis was performed on the co-expressed hub genes to identify potential agents suitable for combating COVID-19. As illustrated in Figure 8, hub genes that are a target of detected FDA-approved drugs were *ISG15* for the Turquoise module of A549 cells and *IL-1β*, *IL-8*, *LIF*, *TNFAIP3*, and *HBEGF* for the Yellow-green module of NHBE cells. However, a large number of these drugs are currently being investigated by several studies to treat SARS-CoV-2 infection [44,46,51,52,53,54,55,56,57,58,59,60]. Thus, we generated a list of 17 novel repurposable drugs that have not yet been explored clinically and need future in vitro and/or in vivo studies to demonstrate their therapeutic properties for the treatment of COVID-19 (Table 2). Overall, our findings provide additional support for drugs that have already reported anti-viral efficacies against COVID-19 and detected promising therapeutic agents that might be potential targets for a closer evaluation.

## 4. Discussion

The infection of SARS-CoV-2 can cause severe pneumonia as well as other complications with significant morbidity and mortality. Our knowledge about the host’s immune interaction with SARS-CoV-2 is restricted, making it more challenging to manage and develop new therapies. A viral infection typically induces massive changes in the transcriptome of the host, resulting in the aberration of host cells’ metabolism and the modulation of the immune response toward enhancing viral replication [61,62]. Using expression data from human lung epithelial cells, including independent biological triplicates of primary human lung epithelium (NHBE) and transformed lung alveolar (A549) cells, we performed deeper transcriptomic analysis to better understand the molecular basis of COVID-19 and identify putative markers. We found that 20 and 42 genes were differentially expressed (FDR < 0.05, and |log_2_FC| ≥ 2) when A549 and NHBE samples of COVID-19 cells were compared to mock-treated controls, respectively. GO functional and KEGG pathway enrichment analysis revealed that these genes belong to the type I interferon signaling pathway, IL-17 signaling pathway [63], cytokine-mediated signaling pathway, and defense response to virus categories, all of which play significant roles in restricting viral infection.

It is noteworthy that we observed a significant increase in *CCL20* (which displays chemotactic activity for lymphocytes), *CXCL8*, *CXCL2*, *CXCL3*, *CXCL5* (as powerful chemoattractants for neutrophils), and *CXCL10* (a regulator of leukocyte chemotaxis) in both cells lines studied, suggesting that the presence of these cells is likely to mediate the induction of numerous chemokines and ILs that contribute to the cytokine storm observed in patients with severe COVID-19, requiring intensive care in hospitals [25]. In addition, our data showed a significantly higher expression level of inflammatory mediators consisting of *G-CSF*, *GMCSF*, *IL-1α*, *IL-1β*, and *IL-6* in response to SARS-CoV-2. In parallel, systemic inflammation and cytokine storms with elevated plasma levels of the *CXCL* family, *G-CSF*, *GMCSF*, *IL-1β*, *IL-8*, and *IL-6* are reported among SARS patients, which are most likely produced by airway epithelial cells as well as a variety of immune cells such as neutrophils, macrophages, lymphocytes, and NK cells [61,64,65,66]. The latest laboratory findings from Wuhan patients also showed that mild COVID-19 patients had elevated levels of *IL-1B*, *IFNγ*, *CXCL10*, and *CCL2*, whereas in severe cases, *G-CSF*, *CXCL10*, *CCL2*, and *CCL3* were elevated [5]. Consistent with our results, a significantly elevated expression of many cytokines was observed in SARS-CoV-2-treated samples compared to mock-treated controls. These findings show that infection with the SARS-CoV-2 virus can result in a cytokine storm that is associated with the severity of the disease. In another study, in patients with SARS disease, *IL-6*, which is needed to regulate the inflammatory response, B-cell differentiation, and antibody development [67], was increased.

Here, we combined machine learning, WGCNA, as well as DEG analysis to develop a reliable data mining approach to extract or filter valuable targets from the transcriptomic data with high dimension, which leads to the construction of a co-expression network based on the verified hub genes in two independent cell lines. It is noteworthy to highlight that several bioinformatic studies have already analyzed transcriptomic changes in the data of lung cell lines infected with COVID-19 using the GSE147507 dataset to identify key genes involved in COVID-19 that could be used as promising targets for drug repositioning. The dysregulation of a majority of these hub genes was reported in recent bioinformatic studies [36]. However, to our knowledge, only an ordinary DEG analysis has already been applied to this dataset without a cell-specific genome-wide method considering gene–gene connectivity patterns and functional gene significance or employing novel big data analytical techniques, including machine learning, which warrants further studies [58,68,69,70,71]. Moreover, the construction of protein–protein interaction (PPI) networks purely based on online databases like STRING and GeneMania may not accurately reflect the condition-specific interactions of the proteins in a given tissue or under certain disease traits [72].

In this regard, Fagone et al. tried to construct a PPI network based on the most differentially expressed genes using the GeneMania database, which identified MX1, IL8, and IFITM1 as the top hub genes as well as BRDeK23875128, SA-792728, and sirolimus, which are the top three potential predicted drugs targeting DEGs. Inconsistent with our results, the functional enrichment of identified DEGs revealed a specific set of biological pathways, including the IL-17 signaling pathway, cytokine-mediated signaling pathway, and defense response to other organisms [73]. Li et al. performed an integrative DEG analysis of the RNA-seq data from different individual studies of COVID-19, including GSE147507, and identified 233 shared differentially expressed genes as well as *LCN2*, *STAT1*, and *UBE2L6* genes as novel candidates in COVID-19 pathogenesis [74]. Similar systemic DEG screening analysis has been used to filter significant up- and down-regulated genes as new COVID-19 repurposing opportunities and finally reported more than 50 drug candidates such as Janus kinase and Bruton kinase inhibitors and corticosteroids capable of combating COVID-19 [75]. Another study deployed a bioinformatics workflow detecting a list of DEGs as gene signatures extracted from RNA-seq data of SARS-CoV-2-infected cell lines of tissues to identify potential drug targets as well as disease signatures. The study reported twenty repurposable drug candidates comprising seven drugs currently undergoing trial, such as Cyclosporine, alongside 12 drugs with anti-viral properties with six drugs possessing especial anti-viral properties against the Coronaviruses family [76]. It is worthwhile to note that another sophisticated molecular profiling work provided by a team from the Krogan lab utilized unbiased, global phosphoproteomics approaches to draw a host–viral protein interactions map upon SARS-CoV-2 infection to identify drug targets with therapeutic potential for COVID-19 treatment. They also estimated transcription factor activities from RNA-seq analysis of different SARS-CoV-2-infected human lung cell lines using the GSE147507 dataset and identified that p38/MAPK transcription factors were among the most highly activated transcription factors induced by SARS-CoV-2 infection, as compared with other transcription factors not related to the p38/MAPK pathway. The results showed that the activation of casein kinase II (CK2) and p38 MAPK signaling pathways as a primary host response over the course of SARS-CoV-2 infection causes dramatic phosphorylation changes on host and viral proteins, pro-inflammatory cytokine production, cytoskeleton remodeling and the shutdown of mitotic kinases, resulting in cell cycle arrest to enhance viral replication. Additionally, the study presented 87 drugs and compounds that were reported to target several host kinase activity states in this process, holding promise to possess anti-viral efficacy for COVID-19 therapies [77]. Inconsistent with our results, these findings further highlight the potential anti-viral role of sunitinib for the appropriate balance of the phosphorylation and activation of kinases and pathways, which may target multiple mechanisms hijacked by SARS-CoV-2 infection (Figure 8).

The use of WGCNA for co-expression analysis does not depend on particular contrasts (differential expression). It may establish associations in the study design between the co-expressed genes and essential factors. Based on our analysis of data in the convenient models of bronchial epithelium cells (NHBE and A549 cells), we created the gene co-expression network using WGCNA and recognized two associated modules. The Turquoise module from A549 samples with 196 genes and the Yellow-green module from NHBE samples with 194 genes were the most correlated modules with SARS-CoV-2 infection. KEGG and GO enrichment analysis of these two modules’ genes further confirmed multiple viral infection-related processes, which were both enriched in acute inflammatory response and defense response to virus categories, which are biologically rational and associated with COVID-19 pathogenicity. One of the remarkable pathways enriched in interesting modules is the regulation of the viral life cycle. The genes in this category have a wide range of roles in the SARS-CoV-2 life cycle, such as facilitating survival, attachment, entry, and replicating the virus particles, which are clear candidate targets of anti-viral drugs [78].

Some of the hub genes identified in our study have recently been reported to be particularly related to COVID-19. For example, the functional overexpression of *MX1* was a significantly mediated anti-viral response both to SARS-CoV and to SARS-CoV-2 [35,36]. Elevated expression levels of *IL-8* and *IL-36G* have been declared to be closely related to COVID-19 severity [11,79].

Whereas a large number of the identified hub genes had a remarkable enrichment in type I interferon (IFN) pathway genes, *PGLYRP4* is known to play a vital IFN-independent role in the regulation of immune responses [80]. *PGLYRP4* is a member of the highly conserved human peptidoglycan recognition proteins (PGLYRPs) family, which recognizes not only bacterial peptidoglycan but also shows direct bactericidal activities against a range of Gram-positive and Gram-negative bacteria [81]. In contrast to this well-known canonical function, *PGLYRP4* has been conclusively demonstrated to display significant inhibitory effects on the expression of pro-inflammatory mediators and tight junction genes, which attenuates host inflammatory defense, and phagocyte recruitment and activation in the lung result in impaired clearance of bacteria and subsequent bacteremia during pneumococcal pneumonia [82]. Intestinally, it has been shown that PGLYRP4 expression could be stimulated by TLR3 ligand polyI: C as a synthetic viral double-stranded RNA (dsRNA), which suggests a potential immunomodulatory role of *PGLYRP4* in response to viral components in addition to its previous anti-bacterial properties [83]. As mentioned earlier, the dysregulation of *PGLYRP4* in the NHBC cell line has been reported by previous in silico studies performing DEG analysis on the GSE147507 dataset [84,85,86,87,88]. Intriguingly, a recent in vitro study showed that treatment with an Hsp90 inhibitor, SNX-5422, exerts promising effects in inhibiting SARS-CoV-2 replication as well as regulating the expression of host factors involved in innate immunity such as *PGLYRP4*. Taken together, these results lead to the hypothesis that *PGLYRP4* up-regulation during SARS-CoV-2 infection may be a novel fundamental mechanism underlying the observed early muted immune response that prevents the successful control of viral spread and, hence, an important target for the development of preventive and therapeutic SARS-CoV-2 interventions [89].

However, the anti-inflammatory effects of *PGLYRP4* demonstrated significant benefits to reduce early inflammatory processes leading to pulmonary damage and hyper inflammation [81]. Strikingly, *PGLYRP4* has been shown to contribute to sphingosine-1-phosphate receptor (S1PR) agonist-mediated disease attenuation during Bordetella pertussis infection, and its beneficial preventive and therapeutic effects on suppressing SARS-CoV-2-related lung damage have been revealed by several studies [90,91,92]. Therefore, these results should be taken with care and warrant further investigations.

*HEPHL1* is another interesting hub gene implicated in the regulation of intracellular iron concentrations exerting an oxidoreductase and ferroxidase activity (Fe^2+^ to Fe^3+^) at the outer cell membrane [80,93]. The importance of this novel member of the multicopper oxidase family for successful iron homeostasis has been demonstrated with *HEPHL1* knockout mouse models as well as the detection of a child with compound heterozygous loss-of-function mutations in *HEPHL1* presenting with an abnormal hair phenotype [94]. Considering the fundamental effects of the iron acquisition, transfer, and storage regulating mechanisms in the proper function of diverse cellular proteins and pathways and that preventing the cytotoxicity induced by the donation of electrons to molecular oxygen results in the generation of toxic free radicals, it is not surprising that iron homeostasis is tightly regulated through various control mechanisms. Moreover, imbalanced iron homeostasis with iron overload has been demonstrated to be implicated during infection with various viruses such as SARS-CoV-2, leading to ARDS and pulmonary fibrosis development [95,96]. Notably, the specific role of *HEPHL1* in regulating cellular iron efflux is mediated by an enzymatic reaction converting Fe (II) to Fe (III), which requires oxygen consumption as an electron acceptor [94]. Thus, one can speculate that perhaps the persistent up-regulation of *HEPHL1* mediated by SARS-CoV-2 replication would participate in gas exchange dysfunction resulting in low oxygen hypoxic conditions and insufficient oxygen supply. Taken together, these findings strengthen the hypothesis that SARS-CoV-2-induced *HEPHL1* dysregulation might promote several detrimental effects in SARS-CoV-2-related lung damage, which not only explains the main clinical manifestations of severe COVID-19 patients, including hypoxemia and dyspnea, but also contributes as the possible underlying mechanism of extracellular microenvironment dysregulation and iron accumulation in the lung tissue, which triggers pulmonary fibrosis and lung function decline following SARS-CoV-2 infection.

As shown in Figure 8, further network-based drug repurposing is calculated and novel and potent drugs targeting SARS-CoV-2 are identified. For instance, danazol (antigonadotropic and anti-estrogen) was discovered in our study and other in silico drug screening studies as a repurposable candidate with demonstrated immunomodulatory properties mediating the attenuation of TNFα-induced IL-8 overexpression [97,98]. Additionally, danazol has been shown to be a candidate agent affecting essential pathways involved in SARS-CoV-2 pathogenesis, including mRNA splicing and ubiquitin-mediated proteolysis pathways [98]. As androgens could be suspected as playing a crucial role in driving both specific SARS-CoV-2 entry receptors’ overexpression and anti-viral immune response suppression, therapeutic interventions with danazol must only be administered with great caution and complete consideration [99]. However, the exact mechanisms that may predispose men, especially aged men, to poorer clinical outcomes and higher mortality rates compared with women remain to be delineated. Collectively, these findings suggest a double-sword role of danazol, which dampened the innate immune response against viral infections, resulting in systemic viral spreading with a poor long-term prognosis on one side but preventing hyperinflammatory syndrome characterized by a fulminant and lethal cytokine storm and multiorgan failure on the other side [99].

Although several studies have already been performed to investigate the potential immunoregulatory roles of anti-IL-1 agents such as anakira for treating severe COVID-19, the use of rilonacept as an IL-1 decoy receptor binding to IL-1β and IL-1α has not yet been explored by preclinical and clinical trial studies [100].

Bisphosphonates such as pamidronic acid, risedronic acid, and tiludronic acid are another promising drug group that has been widely utilized to treat osteoporosis, and similar conditions led to increased bone resorption [101]. Treatment with a member of this family, pamidronic acid, can plausibly ameliorate the disease severity and mortality caused by H1N1 and H5N1 viruses as a result of protective γδ T cells’ expansion in a humanized influenza mouse model, which would be repurposed to enhance γδ T cells’ anti-viral function against SARS-CoV-2 [102]. Notably, studies showed that bisphosphonates have unique immunomodulatory properties, which hold promise for their clinical use to address the symptoms resulting from immune response deterioration and systemic hyper-inflammation that occur in severe COVID-19 [103]. Having gained the various anti-SARS-CoV-2 properties of amino-bisphosphonates such as zoledronic acid in γδ T cell immune response stimulation, dendritic cell modulation, and the disruption of SARS-CoV-2-related endosome trafficking, the repositioning of amino-bisphosphonates has been strongly suggested for treating COVID-19 [104]. Taken together, these findings may offer the bisphosphonate family as potential candidates for drug repurposing, and they could prove to be effective additions to the treatment of COVID, warranting further study.

## 5. Conclusions

To summarize, we used a weighted gene co-expression network analysis to construct a gene co-expression network for COVID-19 and identified a highly correlated hub gene network in A549 and NHBE cell lines. Novel transcriptional signatures of the SARS-CoV-2 virus, including *PGLYRP4* and *HEPHL1*, were detected, and their prospective mechanisms in the pathogenesis of COVID-19 and associated complications were suggested. Additionally, a shortlist of 16 novel candidate repurposable drugs was identified, including drugs that are worthy of further investigation, with the potential to be directly used in clinical trials.

Moving forward, these hub genes may have potential roles in translational medicine and might become promising therapeutic targets.

## Figures and Tables

**Table 1:** Comparison of hub gene expression profiles of interesting modules in the response of SARS-CoV-2, RSV, IAV, and IAV-ΔNS1.

Cell Line	Selected Module	Hub Genes	SARS-CoV-2		RSV		IAV		IAV-ΔNS1	
			logFC	*p*-Value	logFC	*p*-Value	logFC	*p*-Value	logFC	*p*-Value
A549	Turquoise	*MX1*	5.06	3.08 × 10^−168^	5.54	1.76 × 10^−110^	−4.46	ns	-	-
		*IFI6*	4.29	5.12 × 10^−274^	2.92	5.97 × 10^−43^	−0.57	ns	-	-
		*IFIT1*	3.76	4.33 × 10^−163^	5.09	7.98 × 10^−105^	0.03	ns	-	-
		*IFIT3*	1.97	5.28 × 10^−44^	4.99	4.88 × 10^−101^	0.34	ns	-	-
		*OAS1*	1.55	2.74 × 10^−68^	3.10	8.42 × 10^−51^	0.75	0.006989	-	-
		*OAS3*	1.39	3.43 × 10^−57^	2.98	1.27 × 10^−47^	−0.02	ns	-	-
		*ISG15*	4.33	2.78 × 10^−157^	4.52	5.05 × 10^−88^	−0.80	ns	-	-
		*DDX60*	2.30	4.77 × 10^−46^	3.10	1.61 × 10^−38^	−0.06	ns	-	-
		*IRF9*	2.16	1.57 × 10^−84^	1.33	1.61 × 10^−9^	0.46	ns	-	-
		*PARP9*	2.07	1.14 × 10^−75^	3.02	6.60 × 10^−43^	0.42	ns	-	-
NHBE	Yellow-green	*IL36G*	2.72	1.08 × 10^−57^	-		0.88	0.004286	4.12	7.63 × 10^−20^
		*SAA2*	2.41	1.77 × 10^−78^	-	-	0.74	4.02 × 10^−7^	2.69	4.02 × 10^−07^
		*TNIP1*	1.26	1.63 × 10^−37^	-	-	0.01	ns	1.46	9.28 × 10^−8^
		*TNFAIP3*	1.61	2.64 × 10^−49^	-	-	1.41	4.55 × 10^−16^	4.49	1.47 × 10^−22^
		*HEPHL1*	1.30	2.82 × 10^−18^	-	-	1.45	5.47 × 10^−8^	1.56	1.86 × 10^−5^
		*IRAK2*	1.60	2.89 × 10^−22^	-	-	0.97	1.25 × 10^−5^	2.86	3.05 × 10^−23^
		*HBEGF*	1.29	1.07 × 10^−30^	-	-	1.09	0.005477	2.07	8.60 × 10^−24^
		*LIF*	1.30	4.01 × 10^−31^	-	-	0.17	ns	2.89	4.10 × 10^−9^
		*IRF9*	1.25	3.69 × 10^−29^	-	-	1.39	9.52 × 10^−10^	2.75	3.45 × 10^−43^
		*IL1B*	1.06	7.22 × 10^−25^	-	-	−0.04	ns	1.24	5.98 × 10^−9^
		*IL8*	2.32	9.20 × 10^−96^	-	-	-	-	-	-
		*PGLYRP4*	2.02	9.89 × 10^−23^	-	-	1.08	0.088695	1.98	0.004146

**Table 2:** Novel potent drugs with literature-derived anti-viral evidence for COVID-19 drug repositioning.

Modules	Genes	Drugs	Drug Class	PMIDs	DGIdb Score	Anti-Viral Properties
Yellow-green module	IL8	Danazol	Androgen	-	2	-
	IL8	Cetuximab	Antineoplastic, Anti EGFR	-	4	-
	IL8	Aluminum hydroxide	Antacid	-	2	-
	IL8	Talc	Sclerosing agents	-	2	-
	IL8	Alprazolam	Benzodiazepine	12218154	2	IAV
	IL8	Pamidronic acid	Bisphosphonate	21708931	2	IAV
				24154548		EBV
				25220446		
	IL1B	Risedronic acid	Bisphosphonate	-		-
	IL1B	Tiludronic acid	Bisphosphonate	-		-
	IL1B	Rilonacept	Interleukin 1 inhibitor	-		-
	IL1B	Pentamidine	Antiprotozoal	-		-
	IL1B	Glucosamine	Non-steroidal anti-inflammatory and antirheumatic	-		-
	IL1B	Ustekinumab	Anti-IL-12 and IL-23	-		-
	LIF	Capsaicin	Non-narcotic analgesic	32528827		-
				12537981		-
				27574502		-
	LIF	Azacitidine	Antineoplastic, cytosine analogue	32176725		-
	LIF	Paclitaxel	Antineoplastic, Antimicrotubular	31797089	2	-
	HBEGF	Cetuximab	Antineoplastic, Anti EGFR	-	4	-
	HBEGF	Panitumumab	Antineoplastic, Anti EGFR	-		-

PMID: PubMed unique identifier; EBV: Epstein–Barr virus; EGFR: epidermal growth factor receptor; IAV: Influenza A virus.

## Data Availability

All datasets used in this study have been previously published and are accessible via the Gene Expression Omnibus (GEO) website (https://www.ncbi.nlm.nih.gov/geo/, accessed on 12 February 2021) using the GSE accession numbers listed in the Section 2.

## Supplementary Materials

The following are available online at https://www.mdpi.com/article/10.3390/jcm10163567/s1, Table S1: Module colors characterization of A549 dataset, Table S2: Module colors characterization of NHBE dataset, Figure S1: Sample clustering to detect outliers, Figure S2: Heatmap of DEGs between mock-treated and SARS-CoV-2-treated cell lines, Figure S3: Selection of the soft-thresholding powers, Figure S4: Cluster dendrogram and module assignment from WGCNA, Figure S5: Module-trait relationship. Each row corresponds to a module eigengene and column corresponds to SARS-CoV-2 trait, Figure S6: Module-module associations. Eigengene dendrogram and Heatmap plot of the adjacencies in the eigengene network including the state represents the relationships among the modules and disease.

## References

[1] Andersen K.G., Rambaut A., Lipkin W.I., Holmes E.C., Garry R.F. (2020). The proximal origin of SARS-CoV-2. Nat. Med..

[2] Huang C., Wang Y., Li X., Ren L., Zhao J., Hu Y., Zhang L., Fan G., Xu J., Gu X. (2020). Clinical features of patients infected with 2019 novel coronavirus in Wuhan, China. Lancet.

[3] Chan J.F.-W., Yuan S., Kok K.-H., To K.K.-W., Chu H., Yang J., Xing F., Liu J., Yip C.C.-Y., Poon R.W.-S. (2020). A familial cluster of pneumonia associated with the 2019 novel coronavirus indicating person-to-person transmission: A study of a family cluster. Lancet.

[4] Young B.E., Ong S.W.X., Kalimuddin S., Low J.G., Tan S.Y., Loh J., Ng O.-T., Marimuthu K., Ang L.W., Mak T.M. (2020). Epidemiologic features and clinical course of patients infected with SARS-CoV-2 in Singapore. JAMA.

[5] Arabi Y.M., Fowler R., Hayden F.G. (2020). Critical care management of adults with community-acquired severe respiratory viral infection. Intensiv. Care Med..

[6] Cao B., Wang Y., Wen D., Liu W., Wang J., Fan G., Ruan L., Song B., Cai Y., Wei M. (2020). A Trial of Lopinavir–Ritonavir in Adults Hospitalized with Severe COVID-19. N. Engl. J. Med..

[7] Zhou P., Yang X.-L., Wang X.-G., Hu B., Zhang L., Zhang W., Si H.-R., Zhu Y., Li B., Huang C.-L. (2020). A pneumonia outbreak associated with a new coronavirus of probable bat origin. Nature.

[8] Fehr A., Channappanavar R., Perlman S. (2017). Middle East Respiratory Syndrome: Emergence of a Pathogenic Human Coronavirus. Annu. Rev. Med..

[9] Newton A.H., Cardani A., Braciale T.J. (2016). The host immune response in respiratory virus infection: Balancing virus clearance and immunopathology. Semin. Immunopathol..

[10] Xiong Y., Liu Y., Cao L., Wang D., Guo M., Jiang A., Guo D., Hu W., Yang J., Tang Z. (2020). Transcriptomic characteristics of bronchoalveolar lavage fluid and peripheral blood mononuclear cells in COVID-19 patients. Emerg. Microbes Infect..

[11] Ren X., Wang S., Chen X., Wei X., Li G., Ren S., Zhang T., Zhang X., Lu Z., You Z. (2020). Multiple expression assessments of ACE2 and TMPRSS2 SARS-CoV-2 entry molecules in the urinary tract and their associations with clinical manifestations of COVID-19. Infect. Drug Resist..

[12] Ho J.S.Y., Mok B.W.-Y., Campisi L., Jordan T., Yildiz S., Parameswaran S., Wayman J.A., Gaudreault N.N., Meekins D.A., Indran S.V. (2020). Topoisomerase 1 inhibition therapy protects against SARS-CoV-2-induced inflammation and death in animal models. bioRxiv.

[13] Weingarten-Gabbay S., Klaeger S., Sarkizova S., Pearlman L.R., Chen D.-Y., Bauer M.R., Taylor H.B., Conway H.L., Tomkins-Tinch C.H., Finkel Y. (2020). SARS-CoV-2 infected cells present HLA-I peptides from canonical and out-of-frame ORFs. bioRxiv.

[14] Hoagland D.A., Clarke D.J., Moeller R., Han Y., Yang L., Wojciechowicz M.L., Lachmann A., Oguntuyo K.Y., Stevens C., Lee B. (2020). Modulating the transcriptional landscape of SARS-CoV-2 as an effective method for developing antiviral compounds. bioRxiv.

[15] Daniloski Z., Jordan T.X., Wessels H.-H., Hoagland D.A., Kasela S., Legut M., Maniatis S., Mimitou E.P., Lu L., Geller E. (2021). Identification of required host factors for SARS-CoV-2 infection in human cells. Cell.

[16] Wang Y., Liu T., Liu Y., Chen J., Xin B., Wu M., Cui W. (2019). Coronary artery disease associated specific modules and feature genes revealed by integrative methods of WGCNA, MetaDE and machine learning. Gene.

[17] Derakhshani A., Hashemzadeh S., Asadzadeh Z., Shadbad M.A., Rasibonab F., Safarpour H., Jafarlou V., Solimando A.G., Racanelli V., Singh P.K. (2021). Cytotoxic T-Lymphocyte Antigen-4 in Colorectal Cancer: Another Therapeutic Side of Capecitabine. Cancers.

[18] Malik M., Parikh I., Vasquez J.B., Smith C., Tai L., Bu G., Ladu M.J., Fardo D.W., Rebeck G.W., Estus S. (2015). Genetics ignite focus on microglial inflammation in Alzheimer’s disease. Mol. Neurodegener..

[19] Guo S.M., Wang J.X., Li J., Xu F.Y., Wei Q., Wang H.M., Huang H.Q., Zheng S.L., Xie Y.J., Zhang C. (2018). Identification of gene expression profiles and key genes in subchondral bone of osteoarthritis using weighted gene coexpression network analysis. J. Cell. Biochem..

[20] Miao L., Yin R.-X., Pan S.-L., Yang S., Yang D.-Z., Lin W.-X. (2018). Weighted Gene Co-Expression Network Analysis Identifies Specific Modules and Hub Genes Related to Hyperlipidemia. Cell. Physiol. Biochem..

[21] Langfelder P., Horvath S. (2008). WGCNA: An R package for weighted correlation network analysis. BMC Bioinform..

[22] Derakhshani A., Mollaei H., Parsamanesh N., Fereidouni M., Miri-Moghaddam E., Nasseri S., Luo Y., Safarpour H., Baradaran B. (2020). Gene Co-expression Network Analysis for Identifying Modules and Functionally Enriched Pathways in Vitiligo Disease: A Systems Biology Study. Iran. J. Allergy Asthma Immunol..

[23] Daamen A.R., Bachali P., Owen K.A., Kingsmore K.M., Hubbard E.L., Labonte A.C., Robl R., Shrotri S., Grammer A.C., Lipsky P.E. (2021). Comprehensive transcriptomic analysis of COVID-19 blood, lung, and airway. Sci. Rep..

[24] Blanco-Melo D., Nilsson-Payant B.E., Liu W.-C., Uhl S., Hoagland D., Møller R., Jordan T.X., Oishi K., Panis M., Sachs D. (2020). Imbalanced host response to SARS-CoV-2 drives development of COVID-19. Cell.

[25] Gentleman R.C., Carey V.J., Bates D.M., Bolstad B., Dettling M., Dudoit S., Ellis B., Gautier L., Ge Y., Gentry J. (2004). Bioconductor: Open software development for computational biology and bioinformatics. Genome Biol..

[26] Burkart N., Huber M.F. (2021). A survey on the explainability of supervised machine learning. J. Artif. Intell. Res..

[27] Linardatos P., Papastefanopoulos V., Kotsiantis S. (2020). Explainable AI: A Review of Machine Learning Interpretability Methods. Entropy.

[28] Ribeiro M.T., Singh S., Guestrin C. “Why should I trust you?” Explaining the predictions of any classifier. Proceedings of the 22nd ACM SIGKDD International Conference on Knowledge Discovery and Data mining.

[29] Shannon P., Markiel A., Ozier O., Baliga N.S., Wang J.T., Ramage D., Amin N., Schwikowski B., Ideker T. (2003). Cytoscape: A Software Environment for Integrated Models of Biomolecular Interaction Networks. Genome Res..

[30] Cao W., Wu W., Yan M., Tian F., Ma C., Zhang Q., Li X., Han P., Liu Z., Gu J. (2015). Multiple region whole-exome sequencing reveals dramatically evolving intratumor genomic heterogeneity in esophageal squamous cell carcinoma. Oncogenesis.

[31] Asokananthan N., Graham P.T., Fink J., Knight D.A., Bakker A.J., McWilliam A.S., Thompson P.J., Stewart G.A. (2002). Activation of Protease-Activated Receptor (PAR)-1, PAR-2, and PAR-4 Stimulates IL-6, IL-8, and Prostaglandin E2Release from Human Respiratory Epithelial Cells. J. Immunol..

[32] Zaas A.K., Chen M., Varkey J., Veldman T., Hero A.O., Lucas J., Huang Y., Turner R., Gilbert A., Lambkin-Williams R. (2009). Gene expression signatures diagnose influenza and other symptomatic respiratory viral infections in humans. Cell Host Microbe.

[33] Fink K., Martin L., Mukawera E., Chartier S., De Deken X., Brochiero E., Miot F., Grandvaux N. (2013). IFNβ/TNFα synergism induces a non-canonical STAT2/IRF9-dependent pathway triggering a novel DUOX2 NADPH Oxidase-mediated airway antiviral response. Cell Res..

[34] Wein A.N., Dunbar P.R., McMaster S.R., Li Z.-R.T., Denning T., Kohlmeier J.E. (2018). IL-36γ Protects against Severe Influenza Infection by Promoting Lung Alveolar Macrophage Survival and Limiting Viral Replication. J. Immunol..

[35] Leong W., Tan H., Ooi E.E., Koh D., Chow V.T. (2005). Microarray and real-time RT-PCR analyses of differential human gene expression patterns induced by severe acute respiratory syndrome (SARS) coronavirus infection of Vero cells. Microbes Infect..

[36] Pillai P.S., Molony R.D., Martinod K., Dong H., Pang I.K., Tal M., Solis A.G., Bielecki P., Mohanty S., Trentalange M. (2016). Mx1 reveals innate pathways to antiviral resistance and lethal influenza disease. Science.

[37] Kroeker A. (2012). A Proteomic Approach to Discovering Novel Anti-Influenza Mechanisms in Primary Human Airway Epithelial Cells. Ph.D. Thesis.

[38] Barik S. (2013). Respiratory syncytial virus mechanisms to interfere with type 1 interferons. Challenges and Opportunities for Respiratory Syncytial Virus Vaccines.

[39] Imajoh M., Hashida Y., Murakami M., Maeda A., Sato T., Fujieda M., Wakiguchi H., Daibata M. (2012). Characterization of Epstein–Barr virus (EBV) BZLF1 gene promoter variants and comparison of cellular gene expression profiles in Japanese patients with infectious mononucleosis, chronic active EBV infection, and EBV-associated hemophagocytic lymphohistiocytosis. J. Med. Virol..

[40] Zhang Y., Mao D., Roswit W.T., Jin X., Patel A.C., Patel D.A., Agapov E., Wang Z., Tidwell R.M., Atkinson J.J. (2015). PARP9-DTX3L ubiquitin ligase targets host histone H2BJ and viral 3C protease to enhance interferon signaling and control viral infection. Nat. Immunol..

[41] Tatebe K., Zeytun A., Ribeiro R.M., Hoffmann R., Harrod K.S., Forst C.V. (2010). Response network analysis of differential gene expression in human epithelial lung cells during avian influenza infections. BMC Bioinform..

[42] Blanco-Melo D., Nilsson-Payant B., Liu W.-C., Møller R., Panis M., Sachs D., Albrecht R.A., tenOever B.R. (2020). SARS-CoV-2 launches a unique transcriptional signature from in vitro, ex vivo, and in vivo systems. bioRxiv.

[43] Xu Z., Shi L., Wang Y., Zhang J., Huang L., Zhang C., Liu S., Zhao P., Liu H., Zhu L. (2020). Pathological findings of COVID-19 associated with acute respiratory distress syndrome. Lancet Respir. Med..

[44] Jumeau C., Awad F., Assrawi E., Cobret L., Duquesnoy P., Giurgea I., Valeyre D., Grateau G., Amselem S., Bernaudin J.-F. (2019). Expression of SAA1, SAA2 and SAA4 genes in human primary monocytes and monocyte-derived macrophages. PLoS ONE.

[45] Sarma A., Christenson S., Mick E., Deiss T., DeVoe C., Pisco A., Ghale R., Jauregui A., Byrne A., Moazed F. (2021). COVID-19 ARDS is characterized by a dysregulated host response that differs from cytokine storm and is modified by dexamethasone. Res. Sq..

[46] Desai N., Neyaz A., Szabolcs A., Shih A.R., Chen J.H., Thapar V., Nieman L.T., Solovyov A., Mehta A., Lieb D.J. (2020). Temporal and spatial heterogeneity of host response to SARS-CoV-2 pulmonary infection. Nat. Commun..

[47] Hemmat N., Asadzadeh Z., Ahangar N.K., Alemohammad H., Najafzadeh B., Derakhshani A., Baghbanzadeh A., Baghi H.B., Javadrashid D., Najafi S. (2021). The roles of signaling pathways in SARS-CoV-2 infection; lessons learned from SARS-CoV and MERS-CoV. Arch. Virol..

[48] Frohman E.M., Villemarette-Pittman N.R., Cruz R.A., Longmuir R., Rowe V., Rowe E.S., Varkey T.C., Steinman L., Zamvil S.Z., Frohman T.C. (2020). Part II. High-dose methotrexate with leucovorin rescue for severe COVID-19: An immune stabilization strategy for SARS-CoV-2 induced ‘PANIC’ attack. J. Neurol. Sci..

[49] Stegmann K.M., Dickmanns A., Gerber S., Nikolova V., Klemke L., Manzini V., Schloesser D., Bierwirth C., Freund J., Sitte M. (2020). The folate antagonist methotrexate diminishes replication of the coronavirus SARS-CoV-2 and enhances the antiviral efficacy of remdesivir in cell culture models. bioRxiv.

[50] Lee V.S., Chong W.L., Sukumaran S.D., Nimmanpipug P., Letchumanan V., Goh B.H., Lee L.-H., Zain S.M., Abd Rahman N. (2020). Computational screening and identifying binding interaction of anti-viral and anti-malarial drugs: Toward the potential cure for SARS-CoV-2. Prog. Drug Discov. Biomed. Sci..

[51] Galvez J., Zanni R., Galvez-Llompart M. (2020). Drugs Repurposing for Coronavirus Treatment: Computational Study Based On Molecular Topology. Nereis.

[52] Chang Y., Tung Y., Lee K., Chen T., Hsiao Y., Chang H., Hsieh T., Su C., Wang S., Yu J. (2020). Potential Therapeutic Agents for COVID-19 Based on the Analysis of Protease and RNA Polymerase Docking. Preprints.

[53] Contini A. (2020). Virtual screening of an FDA approved drugs database on two COVID-19 coronavirus proteins. Am. Chem. S..

[54] Cava C., Bertoli G., Castiglioni I. (2020). In Silico Discovery of Candidate Drugs against COVID-19. Viruses.

[55] Jeon S., Ko M., Lee J., Choi I., Byun S.Y., Park S., Shum D., Kim S. (2020). Identification of antiviral drug candidates against SARS-CoV-2 from FDA-approved drugs. Antimicrob. Agents Chemother..

[56] Zhang R., Wang X., Ni L., Di X., Ma B., Niu S., Liu C., Reiter R.J. (2020). COVID-19: Melatonin as a potential adjuvant treatment. Life Sci..

[57] Liu X., Li Z., Liu S., Chen Z., Zhao Z., Huang Y.-Y., Zhang Q., Wang J., Shi Y., Xu Y. (2020). Therapeutic effects of dipyridamole on COVID-19 patients with coagulation dysfunction. medRxiv.

[58] Dong L., Hu S., Gao J. (2020). Discovering drugs to treat coronavirus disease 2019 (COVID-19). Drug Discov. Ther..

[59] Zhang R., Li Y., Pan B., Li Y., Liu A., Li X. (2019). Increased expression of hub gene CXCL10 in peripheral blood mononuclear cells of patients with systemic lupus erythematosus. Exp. Ther. Med..

[60] Channappanavar R., Fehr A.R., Vijay R., Mack M., Zhao J., Meyerholz D.K., Perlman S. (2016). Dysregulated type I interferon and inflammatory monocyte-macrophage responses cause lethal pneumonia in SARS-CoV-infected mice. Cell Host Microbe.

[61] Thaker S.K., Ch’Ng J., Christofk H.R. (2019). Viral hijacking of cellular metabolism. BMC Biol..

[62] Pacha O., Sallman M.A., Evans S.E. (2020). COVID-19: A case for inhibiting IL-17?. Nat. Rev. Immunol..

[63] Lu L., Zhang H., Zhan M., Jiang J., Yin H., Dauphars D.J., Li S.-Y., Li Y., He Y.-W. (2020). Preventing Mortality in COVID-19 Patients: Which Cytokine to Target in a Raging Storm?. Front. Cell Dev. Biol..

[64] Okabayashi T., Kariwa H., Yokota S.-I., Iki S., Indoh T., Yokosawa N., Takashima I., Tsutsumi H., Fujii N. (2006). Cytokine regulation in SARS coronavirus infection compared to other respiratory virus infections. J. Med. Virol..

[65] Hemmat N., Derakhshani A., Baghi H.B., Silvestris N., Baradaran B., De Summa S. (2020). Neutrophils, Crucial, or Harmful Immune Cells Involved in Coronavirus Infection: A Bioinformatics Study. Front. Genet..

[66] Zhang Y., Li J., Zhan Y., Wu L., Yu X., Zhang W., Ye L., Xu S., Sun R., Wang Y. (2004). Analysis of Serum Cytokines in Patients with Severe Acute Respiratory Syndrome. Infect. Immun..

[67] Xie T., Han M., Su X., Li H., Chen J., Guo X. (2020). Identification of Hub genes associated with infection of three lung cell lines by SARS-CoV-2 with integrated bioinformatics analysis. J. Cell. Mol. Med..

[68] Cheng L.-C., Kao T.-J., Phan N.N., Chiao C.-C., Yen M.-C., Chen C.-F., Hung J.-H., Jiang J.-Z., Sun Z., Wang C.-Y. (2021). Novel signaling pathways regulate SARS-CoV and SARS-CoV-2 infectious disease. Medicine.

[69] Fang C., Mei J., Tian H., Liou Y.-L., Rong D., Zhang W., Liao Q., Wu N. (2021). CSF3 Is a Potential Drug Target for the Treatment of COVID-19. Front. Physiol..

[70] Cavalli E., Petralia M.C., Basile M.S., Bramanti A., Bramanti P., Nicoletti F., Spandidos D.A., Shoenfeld Y., Fagone P. (2020). Transcriptomic analysis of COVID-19 lungs and bronchoalveolar lavage fluid samples reveals predominant B cell activation responses to infection. Int. J. Mol. Med..

[71] Sevimoglu T., Arga K.Y. (2014). The role of protein interaction networks in systems biomedicine. Comput. Struct. Biotechnol. J..

[72] Fagone P., Ciurleo R., Lombardo S.D., Iacobello C., Palermo C.I., Shoenfeld Y., Bendtzen K., Bramanti P., Nicoletti F. (2020). Transcriptional landscape of SARS-CoV-2 infection dismantles pathogenic pathways activated by the virus, proposes unique sex-specific differences and predicts tailored therapeutic strategies. Autoimmun. Rev..

[73] Li G., Wang J., He X., Zhang L., Ran Q., Xiong A., Wu D., Hu L., Song Q., Zhu D. (2020). An integrative analysis identifying transcriptional features and key genes involved in COVID-19. Epigenomics.

[74] El-Hachem N., Eid E., Nemer G., Dbaibo G., Abbas O., Rubeiz N., Zeineldine S., Matar G.M., Bikorimana J.-P., Shammaa R. (2020). Integrative Transcriptome Analyses Empower the Anti-COVID-19 Drug Arsenal. iScience.

[75] O’Donovan S.M., Imami A., Eby H., Henkel N.D., Creeden J.F., Asah S., Zhang X., Wu X., Alnafisah R., Taylor R.T. (2021). Identification of candidate repurposable drugs to combat COVID-19 using a signature-based approach. Sci. Rep..

[76] Bouhaddou M., Memon D., Meyer B., White K.M., Rezelj V.V., Marrero M.C., Polacco B.J., Melnyk J.E., Ulferts S., Kaake R.M. (2020). The global phosphorylation landscape of SARS-CoV-2 infection. Cell.

[77] Pinto B., Oliveira A.E.R., Singh Y., Jimenez L., Gonçalves A.N.A., Ogava R.L.T., Creighton R., Peron J.P.S., I Nakaya H. (2020). ACE2 Expression Is Increased in the Lungs of Patients With Comorbidities Associated With Severe COVID-19. J. Infect. Dis..

[78] Gong J., Dong H., Xia S.Q., Huang Y.Z., Wang D., Zhao Y., Liu W., Tu S., Zhang M., Wang Q. (2020). Correlation Analysis Between Disease Severity and Inflammation-related Parameters in Patients with COVID-19 Pneumonia. medRxiv.

[79] Belinky F., Nativ N., Stelzer G., Zimmerman S., Stein T.I., Safran M., Lancet R. (2015). PathCards: Multi-source consolidation of human biological pathways. Database.

[80] Skerry C., Goldman W.E., Carbonetti N.H. (2019). Peptidoglycan Recognition Protein 4 Suppresses Early Inflammatory Responses to Bordetella pertussis and Contributes to Sphingosine-1-Phosphate Receptor Agonist-Mediated Disease Attenuation. Infect. Immun..

[81] Dabrowski A.N., Shrivastav A., Conrad C., Komma K., Weigel M., Dietert K., Gruber A.D., Bertrams W., Wilhelm J., Schmeck B. (2019). Peptidoglycan Recognition Protein 4 Limits Bacterial Clearance and Inflammation in Lungs by Control of the Gut Microbiota. Front. Immunol..

[82] Ma P., Wang Z., Pflugfelder S.C., Li D.-Q. (2010). Toll-like receptors mediate induction of peptidoglycan recognition proteins in human corneal epithelial cells. Exp. Eye Res..

[83] Moni M.A., Quinn J.M., Sinmaz N., Summers M.A. (2021). Gene expression profiling of SARS-CoV-2 infections reveal distinct primary lung cell and systemic immune infection responses that identify pathways relevant in COVID-19 disease. Brief. Bioinform..

[84] Chandrashekar D.S., Manne U., Varambally S. (2020). Comparative transcriptome analyses reveal genes associated with SARS-CoV-2 infection of human lung epithelial cells. bioRxiv.

[85] Al Heialy S., Hachim M.Y., Senok A., Gaudet M., Tayoun A.A., Hamoudi R., Alsheikh-Ali A., Hamid Q. (2020). Regulation of Angiotensin- Converting Enzyme 2 in Obesity: Implications for COVID-19. Front. Physiol..

[86] Karakurt H.U., Pınar P. (2020). Integration of transcriptomic profile of SARS-CoV-2 infected normal human bronchial epi-thelial cells with metabolic and protein-protein interaction networks. Turk. J. Biol..

[87] Kang K., Kim H.H., Choi Y. (2020). Tiotropium Is Predicted to Be a Promising Drug for COVID-19 Through Transcriptome-Based Comprehensive Molecular Pathway Analysis. Viruses.

[88] Goswami R., Russell V.S., Tu J.J., Hughes P.F., Kelly F., Langel S.N., Steppe J., Palmer S.M., Haystead T., Blasi M. (2021). Oral Hsp90 inhibitor, SNX-5422, attenuates SARS-CoV-2 replication and dampens inflammation in airway cells. SSRN.

[89] Catanzaro M., Fagiani F., Racchi M., Corsini E., Govoni S., Lanni C. (2020). Immune response in COVID-19: Addressing a phar-macological challenge by targeting pathways triggered by SARS-CoV-2. Signal Transduct. Target. Ther..

[90] Meacci E., Garcia-Gil M., Pierucci F. (2020). SARS-CoV-2 infection: A role for S1P/S1P receptor signaling in the nervous system?. Int. J. Mol. Sci..

[91] Zrzavy T., Wimmer I., Rommer P.S., Berger T. (2020). Immunology of COVID-19 and disease-modifying therapies: The good, the bad and the unknown. Eur. J. Neurol..

[92] Cronin S.J.F., Woolf C.J., Weiss G., Penninger J.M. (2019). The Role of Iron Regulation in Immunometabolism and Immune-Related Disease. Front. Mol. Biosci..

[93] Sharma P., Reichert M., Lu Y., Markello T.C., Adams D.R., Steinbach P.J., Fuqua B.K., Parisi X., Kaler S.G., Vulpe C.D. (2019). Biallelic HEPHL1 variants impair ferroxidase activity and cause an abnormal hair phenotype. PLoS Genet..

[94] Dalamaga M., Karampela I., Mantzoros C.S. (2020). Commentary: Could iron chelators prove to be useful as an adjunct to COVID-19 Treatment Regimens?. Metabolism.

[95] Drakesmith H., Prentice A. (2008). Viral infection and iron metabolism. Nat. Rev. Microbiol..

[96] Horie S., Harada T., Mitsunari M., Taniguchi F., Iwabe T., Terakawa N. (2005). Progesterone and progestational compounds attenuate tumor necrosis factor alpha–induced interleukin-8 production via nuclear factor kappaB inactivation in endometriotic stromal cells. Fertil. Steril..

[97] Barh D., Tiwari S., Weener M.E., Azevedo V., Góes-Neto A., Gromiha M.M., Ghosh P. (2020). Multi-omics-based identification of SARS-CoV-2 infection biology and candidate drugs against COVID-19. Comput. Biol. Med..

[98] Xu Y., Liu S., Zhang Y., Zhi Y. (2020). Does hereditary angioedema make COVID-19 worse?. World Allergy Organ. J..

[99] Soy M., Keser G., Atagündüz P., Tabak F., Atagündüz I., Kayhan S. (2020). Cytokine storm in COVID-19: Pathogenesis and overview of anti-inflammatory agents used in treatment. Clin. Rheumatol..

[100] Cremers S., Papapoulos S. (2011). Pharmacology of bisphosphonates. Bone.

[101] Yazdanifar M., Mashkour N., Bertaina A. (2020). Making a case for using γδ T cells against SARS-CoV-2. Crit. Rev. Microbiol..

[102] Fujimura T., Kambayashi Y., Furudate S., Kakizaki A., Aiba S. (2013). Immunomodulatory Effect of Bisphosphonate Risedronate Sodium on CD163+ Arginase 1+ M2 Macrophages: The Development of a Possible Supportive Therapy for Angiosarcoma. Clin. Dev. Immunol..

[103] Brufsky A., Marti J.L.G., Nasrazadani A., Lotze M.T. (2020). Boning up: Amino-bisphophonates as immunostimulants and endosomal disruptors of dendritic cell in SARS-CoV-2 infection. J. Transl. Med..

[104] Karami H., Derakhshani A., Fereidouni M., Miri-Moghaddam E., Baradaran B., Silvestris N., Paradiso A.V., Safarpour H., De Summa S. (2020). Transcriptional analysis of lung epithelial cells using WGCNA revealed the role of IRF9 and IFI6 genes in SARS-CoV-2 pathogenicity. Res. Sq..

