# Supplementary material for: Weighted Gene Co-Expression Network Analysis Combined with Machine Learning Validation to Identify Key Modules and Hub Genes Associated with SARS-CoV-2 Infection
Source: J Clin Med. 2021 Aug 13;10(16):3567. doi: 10.3390/jcm10163567 (PMC8397209; doi:10.3390/jcm10163567)
Supplement: Supplementary file 1 [file jcm-10-03567-s001.zip › jcm-1289830-supplementary.pdf]

Supplementary Materials

Table S1. Module colors characterization of A549 dataset.

#	Module Colors‡	Number of Genes‡	Correlation‡	*p*-value‡
1‡	Turquoise‡	196‡	1‡	1.00×10^{-5}‡
2‡	Grey60‡	138‡	0.72‡	0.1‡
3‡	Brown4‡	1313‡	0.53‡	0.3‡
4‡	Darkturquoise‡	111‡	0.44‡	0.4‡
5‡	Thistle2‡	41‡	0.4‡	0.4‡
6‡	Red‡	217‡	0.32‡	0.5‡
7‡	Skyblue3‡	47‡	0.22‡	0.7‡
8‡	Salmon4‡	91‡	−0.1‡	0.9‡
9‡	Darkolivegreen‡	216‡	−0.16‡	0.8‡
10‡	Saddlebrown‡	56‡	−0.16‡	0.8‡
11‡	Mediumpurple2‡	30‡	−0.19‡	0.7‡
12‡	Coral2‡	118‡	−0.28‡	0.6‡
13‡	Lightpink4‡	35‡	−0.3‡	0.6‡
14‡	Cyan‡	201‡	−0.31‡	0.5‡
15‡	Palevioletred3‡	82‡	−0.33‡	0.5‡
16‡	Maroon‡	37‡	−0.54‡	0.3‡
17‡	Plum‡	188‡	−0.67‡	0.1‡
18‡	Darkslateblue‡	233‡	−0.77‡	0.07‡
19‡	Lightsteelblue1‡	529‡	−0.86‡	0.03‡
20	Darkorange2	121	−0.89	0.02

Table S2. Module colors characterization of NHBE dataset.

#	Module Colors‡	Number of Genes‡	Correlation‡	*p*-value‡
1‡	Yellowgreen‡	198‡	1‡	2.00×10^{-5}‡
2‡	Violet‡	57‡	0.72‡	0.1‡
3‡	Black‡	175‡	0.65‡	0.2‡
4‡	Cyan‡	68‡	0.4‡	0.4‡
5‡	Coral1‡	68‡	0.26‡	0.6‡
6‡	Darkred‡	112‡	0.19‡	0.7‡
7‡	Plum1‡	242‡	0.14‡	0.8‡
8‡	Floralwhite‡	425‡	0.1‡	0.8‡
9‡	Darkorange2‡	46‡	0.075‡	0.9‡
10‡	Indianred4‡	88‡	0.058‡	0.9‡
11‡	Yellow4‡	33‡	-0.066‡	0.9‡
12‡	Lightcyan1‡	48‡	-0.075‡	0.9‡
13‡	Darkseagreen4‡	74‡	−0.18‡	0.7‡
14‡	Sienna3‡	53‡	−0.28‡	0.6‡
15‡	Thistle2‡	132‡	−0.36‡	0.5‡
16‡	Lavenderblush3‡	38‡	−0.38‡	0.5‡
17‡	Blue‡	154‡	−0.47‡	0.4‡
18‡	Bisque4‡	214‡	−0.56‡	0.2‡
19‡	Darkgrey‡	120‡	−0.6‡	0.2‡
20‡	Darkolivegreen‡	841‡	−0.65‡	0.2‡
21‡	Brown2‡	184‡	−0.74‡	0.09‡
22‡	Pink‡	206‡	−0.76‡	0.08‡
23	Darkgreen	186	−0.82	0.05
24	Coral2	238	−0.92	0.01

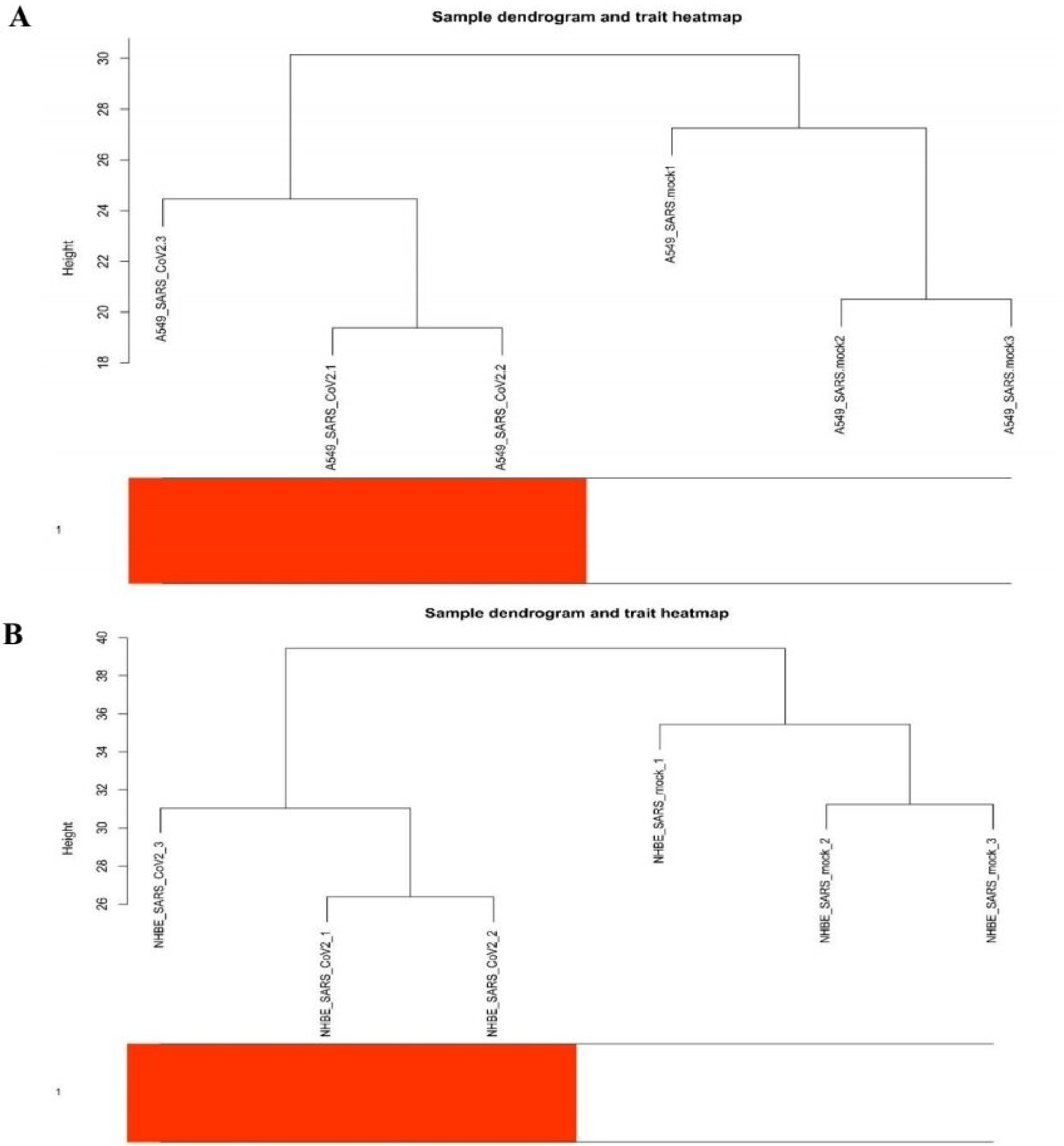

Figure S1. Sample clustering to detect outliers. (**A**) A549 dataset and (**B**) NHBE dataset. The color is proportional to the pathological stage (Red=infected samples and white=mock treated samples).

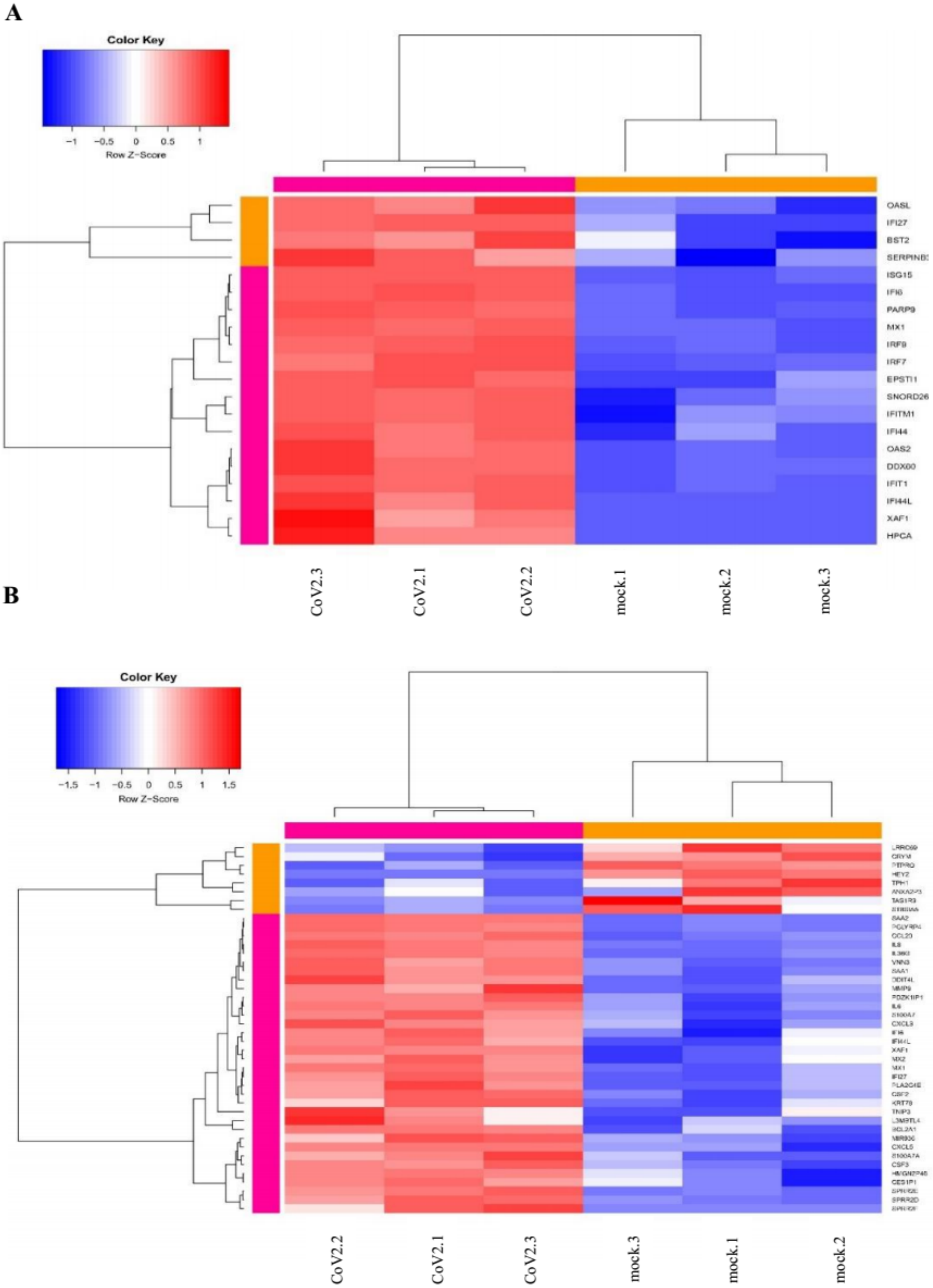

Figure S2. Heatmap of DEGs between mock-treated and SARS-CoV-2-treated cell lines. (**A**) A549 dataset and (**B**) NHBE dataset.

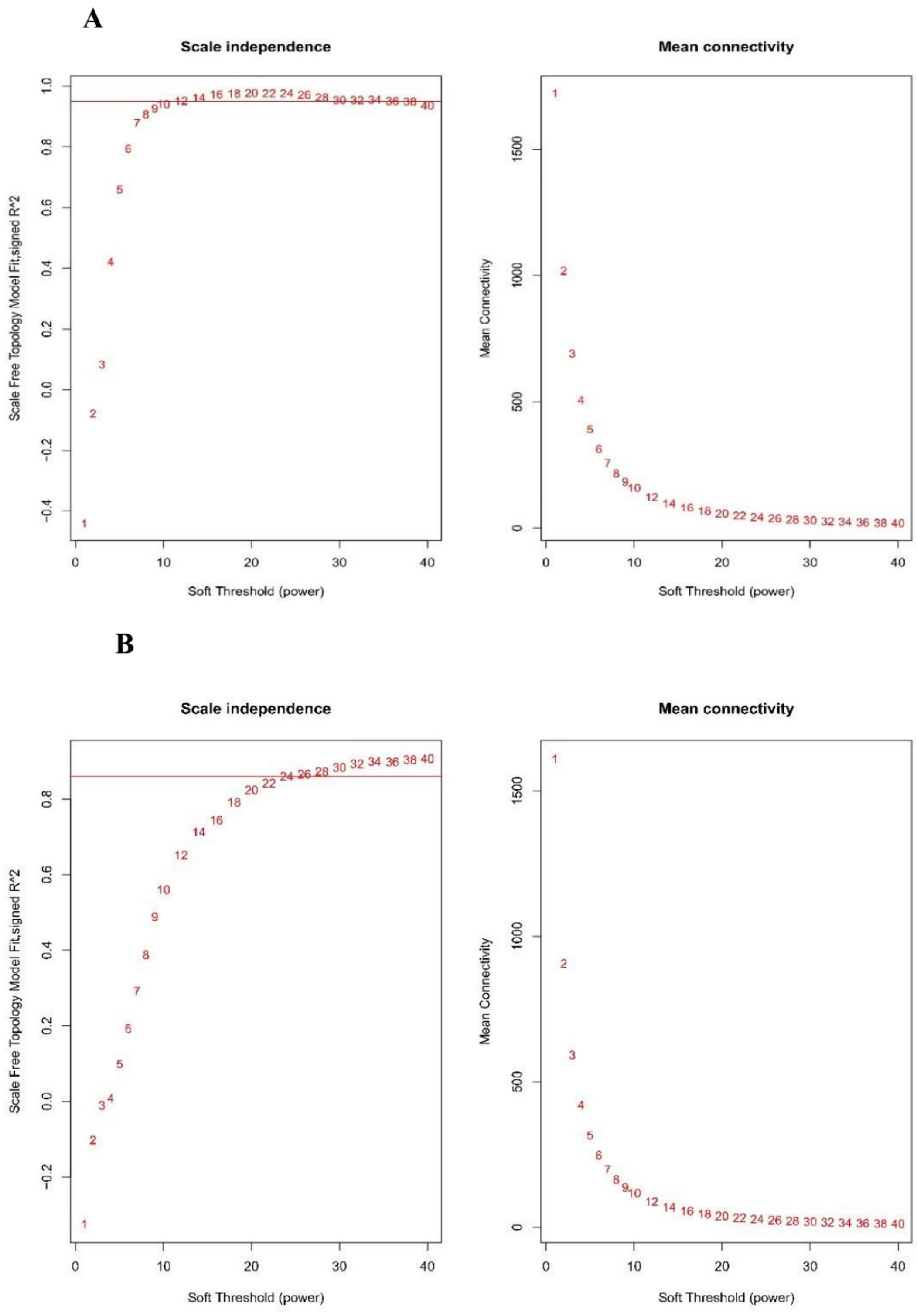

Figure S3. Selection of the soft-thresholding powers. (**A**) A549 dataset and (**B**) NHBE dataset. The left panel shows the Scheme 9. and 22 for A549 and NHBE datasets, respectively for the next analysis.

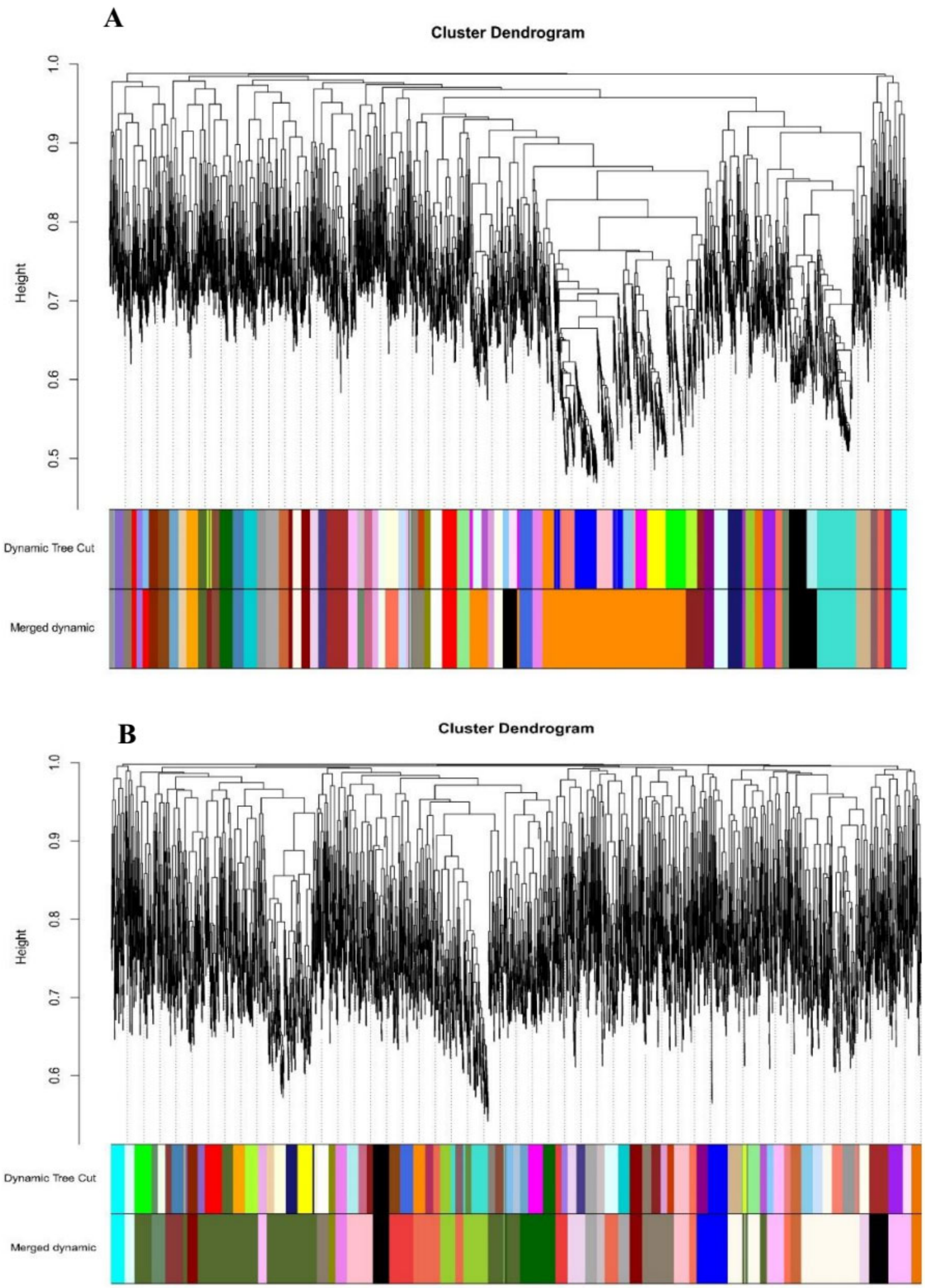

Figure S4. Cluster dendrogram and module assignment from WGCNA. The branches correspond to highly interconnected groups of genes. Colors in the horizontal bar represent the modules. (**A**) A549 dataset and (**B**) NHBE dataset.

A

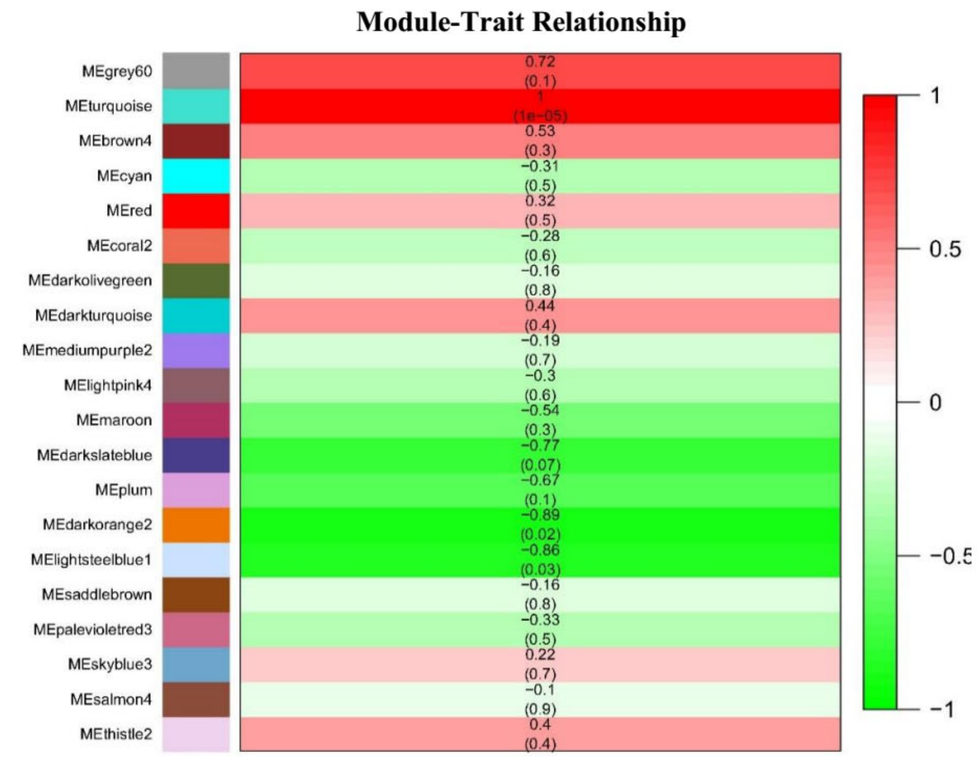

B

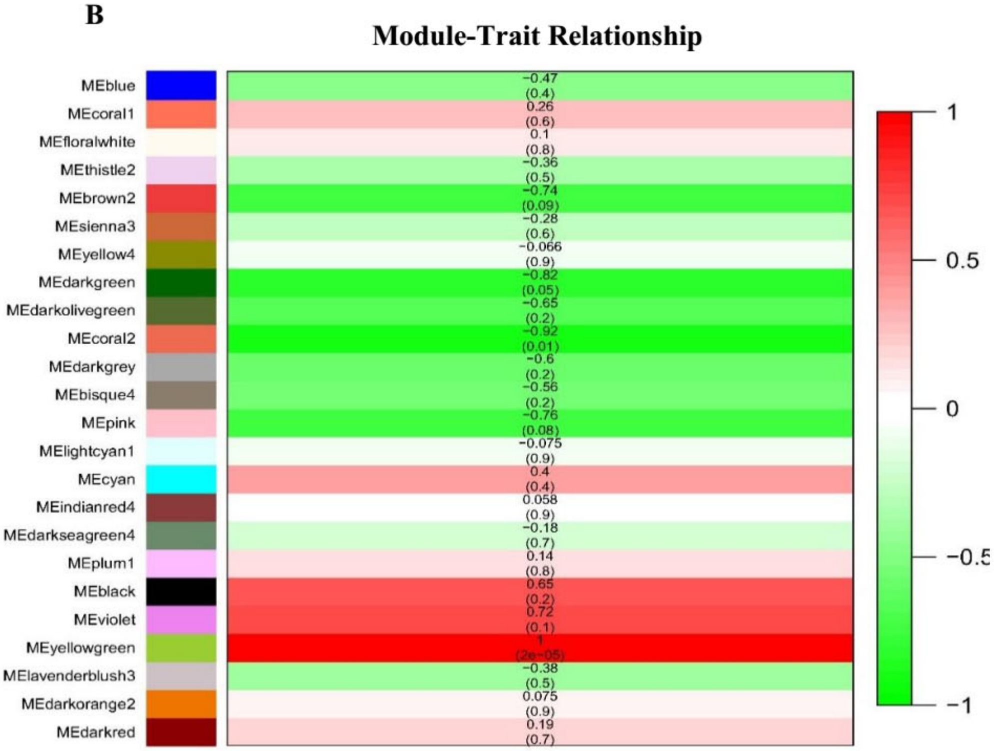

Figure S5. Module-trait relationship. Each row corresponds to a module eigengene and column corresponds to SARS-CoV-2 trait. Numbers in each cell represent the corresponding correlation and p value. (**A**) A549 dataset and (**B**) NHBE dataset.

A

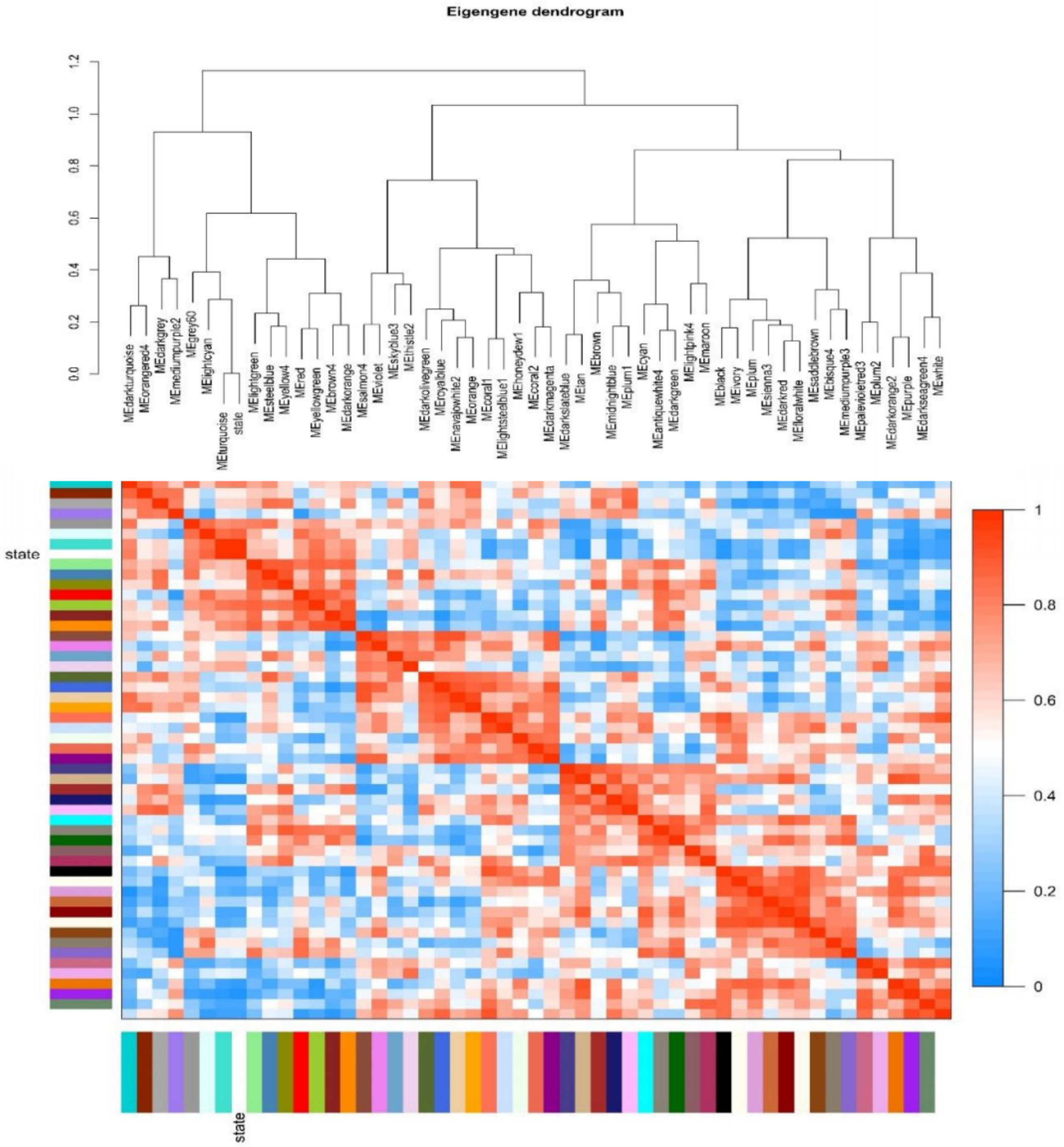
Eigengene dendrogram
1.2
1.0
0.8
0.6
0.4
0.2
0.0
MEdarkturquoise
MEorangered4
MEdarkgrey
MEmediumpurple2
MEgrey60
MElightcyan
MEturquoise
state
MElightgreen
MEsteelblue
MEyellow4
MEred
MEyellowgreen
MEbrown4
MEdarkorange
MEsalmon4
MEviolet
MEskyblue3
MEthistle2
MEdarkolivegreen
MEroyalblue
MEnavajowhite2
MEorange
MEcoral1
MElightsteelblue1
MEhoneydew1
MEcoral2
MEdarkmagenta
MEdarkslateblue
MEtan
MEbrown
MEmidnightblue
MEplum1
MEcyan
MEantiquewhite4
MEdarkgreen
MElightpink4
MEmaroon
MEblack
MEivory
MEplum
MEsienna3
MEdarkred
MEfloralwhite
MEsaddlebrown
MEbisque4
MEmediumpurple3
MEpalevioletred3
MEplum2
MEdarkorange2
MEpurple
MEdarkseagreen4
MEwhite
state
1
0.8
0.6
0.4
0.2
0
state

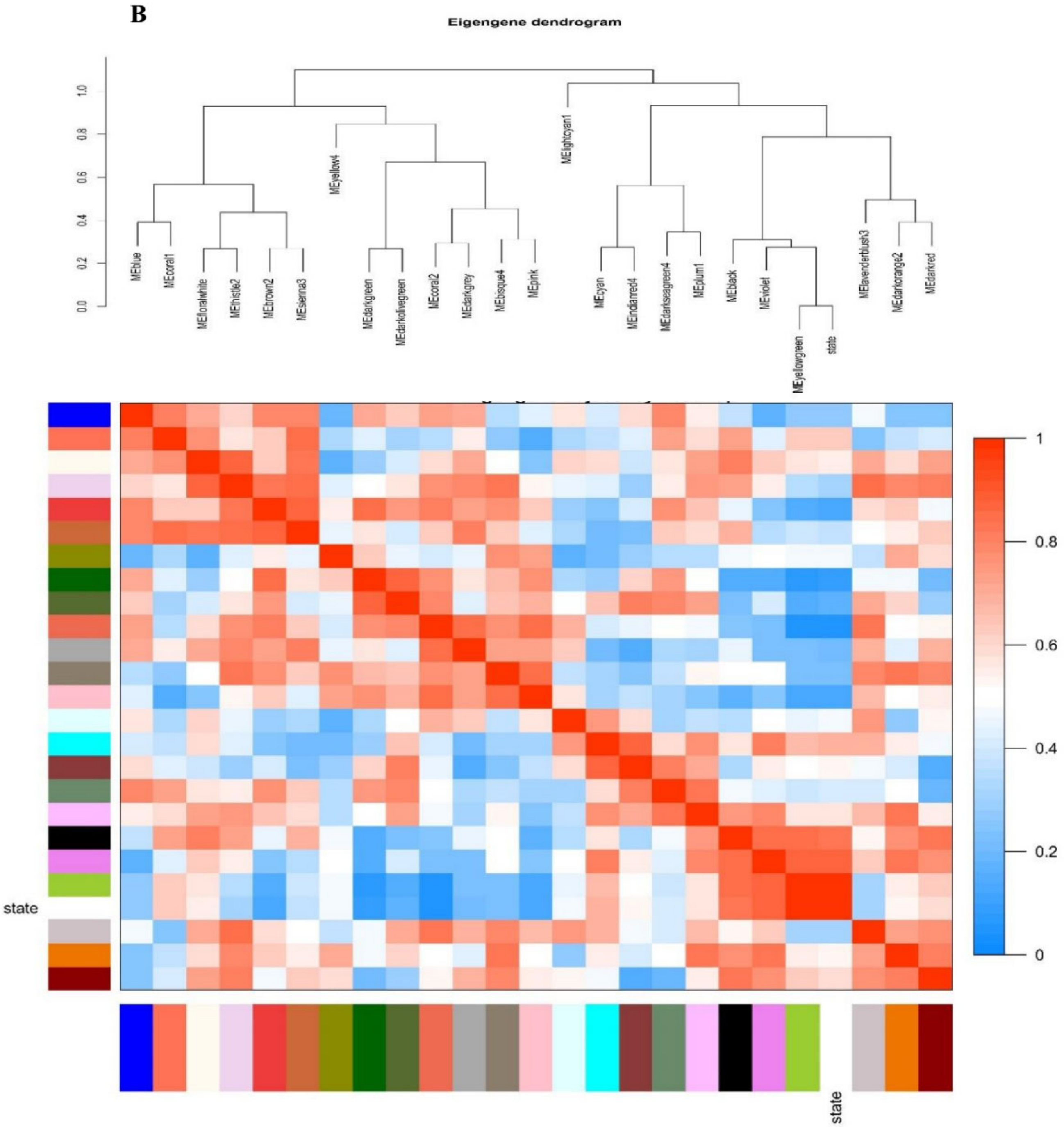

Figure S6. Module-module associations. Eigengene dendrogram and Heatmap plot of the adjacencies in the eigengene network including the state represents the relationships among the modules and disease. (**A**) A549 dataset and (**B**) NHBE dataset.
